# Simultaneous Assessment of Soil Microbial Community Structure and Function through Analysis of the Meta-Transcriptome

**DOI:** 10.1371/journal.pone.0002527

**Published:** 2008-06-25

**Authors:** Tim Urich, Anders Lanzén, Ji Qi, Daniel H. Huson, Christa Schleper, Stephan C. Schuster

**Affiliations:** 1 Centre of Geobiology, Department of Biology, University of Bergen, Bergen, Norway; 2 Department of Genetics in Ecology, Vienna Ecology Center, University of Vienna, Vienna, Austria; 3 Computational Biology Unit, Bergen Center for Computational Science, University of Bergen, Bergen, Norway; 4 Center for Comparative Genomics and Bioinformatics, Pennsylvania State University, State College, Pennsylvania, United States of America; 5 Center for Bioinformatics, Tübingen University, Tübingen, Germany; University of Wyoming, United States of America

## Abstract

**Background:**

Soil ecosystems harbor the most complex prokaryotic and eukaryotic microbial communities on Earth. Experimental approaches studying these systems usually focus on either the soil community's taxonomic structure or its functional characteristics. Many methods target DNA as marker molecule and use PCR for amplification.

**Methodology/Principal Findings:**

Here we apply an RNA-centered meta-transcriptomic approach to simultaneously obtain information on both structure and function of a soil community. Total community RNA is random reversely transcribed into cDNA without any PCR or cloning step. Direct pyrosequencing produces large numbers of cDNA rRNA-tags; these are taxonomically profiled in a binning approach using the MEGAN software and two specifically compiled rRNA reference databases containing small and large subunit rRNA sequences. The pyrosequencing also produces mRNA-tags; these provide a sequence-based transcriptome of the community. One soil dataset of 258,411 RNA-tags of ∼98 bp length contained 193,219 rRNA-tags with valid taxonomic information, together with 21,133 mRNA-tags. Quantitative information about the relative abundance of organisms from all three domains of life and from different trophic levels was obtained in a single experiment. Less frequent taxa, such as soil Crenarchaeota, were well represented in the data set. These were identified by more than 2,000 rRNA-tags; furthermore, their activity *in situ* was revealed through the presence of mRNA-tags specific for enzymes involved in ammonia oxidation and CO_2_ fixation.

**Conclusions/Significance:**

This approach could be widely applied in microbial ecology by efficiently linking community structure and function in a single experiment while avoiding biases inherent in other methods.

## Introduction

Soils cover almost all of the terrestrial area on Earth and have an indispensable ecological function in the global cycles of carbon, nitrogen and sulfur. Due to their physico-chemical complexity with many micro-niches, they teem with bio-diversity, both phylogenetically and functionally. A single gram of soil has been estimated to contain thousands to millions of different bacterial, archaeal and eukaryotic species [1], [2] interwoven in extremely complex food webs. Communities of soil microbes carry out a multitude of small-scale processes that underlie many environmentally important functions. However, the explicit functional and ecological roles of individual taxa remain uncertain because most microbes withstand laboratory cultivation [3], [4]. Therefore the most basic questions in microbial ecology “who is out there?” and “what are they doing?” are still often unanswered for many environments and for many microbial taxa. Ideally, especially the second question requires simultaneously information about the identity of taxa within a community and about functional processes performed. While soils seem to harbor the most complex microbial communities, these considerations apply to many other environments as well, like e.g. oceans and sediments.

With metagenomic technologies new dimensions in the characterization of complex microbial communities have been reached. Large scale shotgun sequencing approaches allow the discovery of many novel genes found in the environments independent of cultivation efforts [5]–[8]. Sequencing of large genomic inserts that contain phylogenetic “anchors” allows a direct link to the microbial taxon. However, in almost all of the metagenomic studies, a separate accompanying molecular typing method-usually based on PCR-amplified 16S rRNA genes-is needed to characterize the gene discoveries in the context of the microbial phylogenetic diversity of the sample [7]–[9].

These PCR-based typing methods-though very powerful, in particular when combined with the novel pyro-sequencing technology-[10], [11] have some well-known drawbacks: (1) bias is introduced by primers and/or exponential amplification; (2) simultaneous quantitative assessment of all three domains of life is impossible and (3) persistence of free DNA can bias the measurement of community responses to environmental changes.

Furthermore, DNA-based metagenomic and diversity studies do not allow us to draw conclusions on the expression state of the genes and therefore the functional role of genes or organisms in the investigated environment remains uncertain. In analogy to postgenomic studies of cultivated organisms, a logical next step in the metagenomic area therefore includes meta-transcriptomic technology.

First attempts to study the transcription of genes in environmental samples have been performed. They involved specific purification steps to selectively enrich for mRNA of bacteria or eukarya by depleting ribosomal RNA or enriching for polyA-tailed mRNA, respectively [12], [13]. A more large scale (pyro-)sequencing approach was recently adapted for use with bacterial and archaeal mRNA from an environmental marine sample [14]. It involved an *in vitro* amplification step to maintain small sample size and fast preparation.

In order to overcome some of the limitations of the approaches mentioned above, we explore here the possibility to analyse the total RNA pool of a community, as it is naturally enriched not only in functionally but also taxonomically relevant molecules, i.e. mRNA and rRNA, respectively.

This offers the opportunity to link community structure and function in a single experiment, and to reach beyond the community's genomic potential as assessed in DNA-based methods, towards its *in situ* activity. Furthermore, the use of total RNA avoids extensive cleaning or amplification steps of mRNA molecules, enabling fast preparation even from difficult samples, such as soils, which are notoriously enriched for humic acids and other substances inhibiting molecular biological applications.

We have published initial results of the approach earlier in the context of characterizing the transcriptional activity of particular organisms from soil [15]. Here we present and validate the “double-RNA” approach for in-depth characterization of a soil microbial community by studying mRNA and rRNA molecules simultaneously from the same sample. Our approach ensures (i) fast preparation of the RNA in the light of the short half life of mRNA molecules, it avoids (ii) biases introduced by PCR or cloning procedures and (iii) obtains qualitative and quantitative information simultaneously of genes from organisms of all three domains of life, the archaea, bacteria and eukarya. Since highly parallel sequencing was used for this approach, which produced many, but short reads (100 bp), we have set up an appropriate bioinformatic analysis pipeline that allows to reliably extract in-depth functional and taxonomic information from this dataset.

## Results and Discussion

### Community profiling based on SSU and LSU rRNA-tags

**Figure 1:**
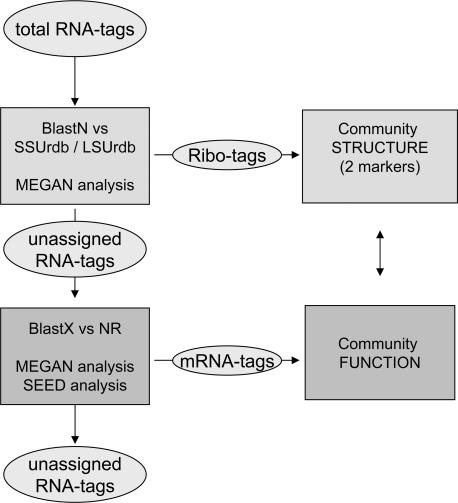
Overview about the double RNA analysis pipeline. Refer to text for details.

The sandy soil ecosystem studied here stems from a conservation area and is comparably poor in nutrients and of neutral pH. It has been the subject of a variety of studies. Many molecular data are therefore available which facilitate result interpretation [15]–[20]. We generated cDNA from the transcriptome of this soil community by a non-targeted approach applying random-hexamer primed reverse transcription on total RNA. The cDNA was subjected directly to pyrosequencing without any prior PCR or cloning steps (see materials and methods for details). Several potentially biasing steps were therefore avoided. One experiment resulted in 258,411 RNA-derived sequence tags of ∼98 bp. We set up a two-step analysis process to identify rRNA- and mRNA-derived tags (“ribo-tags” and “mRNA-tags”, respectively) for efficiently and reliably obtaining taxonomic and functional information (Fig. 1). In the first step, all RNA-tags were compared against a small subunit and a large subunit rRNA reference database (SSUrdb and LSUrdb, respectively) that we compiled from publicly available sources (SILVA[21], RDP-II[22], Genbank nucleotide; see materials and methods for details). These two databases contain sequences from all three domains of life (SSUrdb: 137,160 sequences; LSUrdb: 6,247 sequences; see Supplement Table S1 in Supporting Information, SI). This approach enables the first simultaneous taxonomic analysis of communities based on the two most commonly used taxonomic marker molecules. The output file was analyzed with the program MEGAN[23]. In the second stage, all unassigned RNA-tags were compared against the Genbank non-redundant protein database to identify mRNA-tags.

The validity of our taxonomic analysis was tested with two simulated datasets composed of 43 small or large subunit rRNAs from bacterial (32), archaeal (5) and eukaryotic (6) representatives (see Supplement Methods and Results S1 in SI for details). Both databases, though very different in size and sequence composition, produced a similarly high taxonomic resolution power not only at the taxonomic level of domain and phylum, but also at the level of order for most of the species tested (Tables S3 and S4 in SI give details). These results also showed that neither reference database introduced a major artificial “community” shift; rather, both correctly reflected the test set's “community” structure. In addition, none of the ribo-tags were taxonomically wrongly assigned. Even with a ribo-tag length of 100 bp the power of resolution sufficed for our approach (this has also been demonstrated by other simulations, e.g. [24], [25]). Additionally, we simulated a more natural situation by removing all reference sequences similar to test rRNAs from the databases, according to similarity distributions of ribo-tags from our soil dataset against the reference databases (see Supplement Methods and Results S1 in SI for details). The identified thresholds reflected the median similarity of SSU and LSU ribo-tags (98% and 93% respectively; see Figures S1 and S2 in SI) to their best BLAST match in the reference databases. No significant decrease in taxonomic resolution was observed for the SSUrdb and no ribo-tag was incorrectly assigned (Table S5 and Figure S3 in SI). The much smaller LSUrdb assigned 5% of the ribo-tags incorrectly (Table S5 and Figure S4 in SI). When removing all reference sequences ≥86% similar to test sequences from the SSUrdb (to simulate a situation reflecting to the lowest decile similarity for SSU ribo-tags observed in the soil dataset), 5% of the ribo-tags were incorrectly assigned (Table S5 in SI). These results indicated a robust performance of the taxonomic binning approach. Encouragingly, the taxonomic resolution power will only improve further as (1) read lengths increase –250 bp are now possible, and 450 bp will soon be achievable; and (2) rRNA reference databases compile ever more sequences.

**Figure 2:**
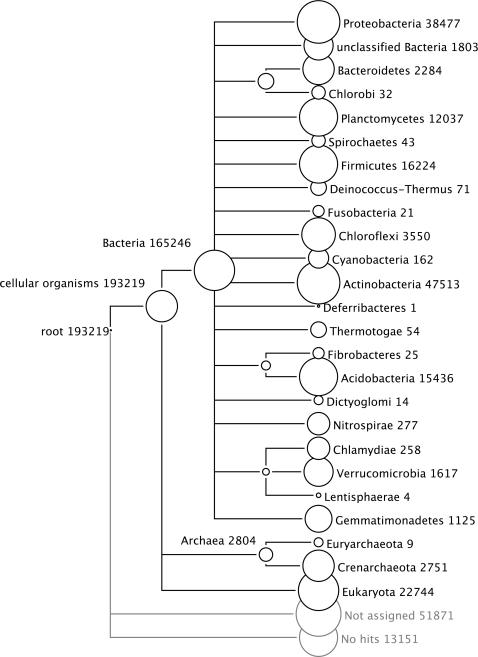
In-depth taxonomic community profiling based on SSU and LSU rRNA. MEGAN tree with the taxonomic affiliation of ribo-tags identified by BLASTN of all RNA-tags against our SSUrdb and LSUrdb according to the NCBI taxonomy. The numbers and sizes of the circles at the tree nodes refer to the ribo-tags affiliated with the respective taxon (absolute cutoff: BLASTN bit score 86, relative cutoff 10% of BLASTN top hit).

The soil dataset contained 193,219 RNA-tags which had significant BLAST hits against the rRNA reference databases (Table 1 and Fig 2). This dataset–which was generated without any PCR or cloning steps–was two to three orders of magnitude larger than traditional rDNA clone libraries, and was also far larger than recently published soil-derived pyro-sequencing datasets [26],

**Table 1:** Statistics of soil community RNA-tag analysis.

	No. of RNA-tags	% of RNA-tags
Total RNA-tags	258,411	100
Total ribo-tags	193,219	74.8
SSU ribo-tags	99,061	38.3
LSU ribo-tags	94,165	36.4
mRNA-tags	21,133	8.2
unassigned RNA-tags	44,059	17.0

Note that the number of total ribo-tags differs from the sum of SSU and LSU ribo-tags. Seven ribo-tags contained regions with significant similarity against both databases (not shown). These putative chimeras were likely produced from SSU and LSU derived cDNA fragments during second-strand cDNA synthesis.

**Figure 3:**
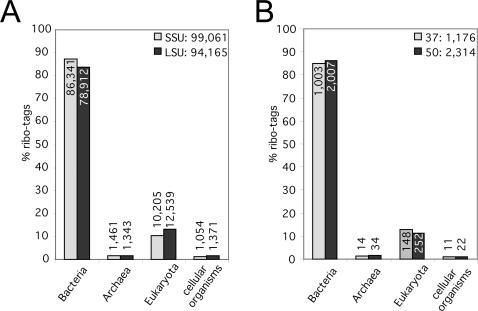
a, Relative ribo-tag distribution of the different cellular domains of life in the SSUrdb and LSUrdb. Absolute numbers are additionally given. Both reference databases report similar fractions, with bacteria-derived ribo-tags being most prominent. Note that approximately 1% of the ribo-tags are not affiliated to any domain by the reference databases. b, Comparison of relative ribo-tag distribution from two independent cDNA syntheses at 37°C (n = 4141 RNA-tags) and 50°C (n = 1985 RNA-tags) performed on the same RNA pool. Values represent the mean of SSU and LSU ribo-tags.

The SSUrdb and LSUrdb reported very similar relative community proportions on the domain level, with 10.3% and 13.3% of ribo-tags stemming from Eukarya, 87.2% and 83.8% from Bacteria and 1.5% and 1.4% from Archaea, respectively (Fig. 3a), which shows the reliability of our analytical approach. We further confirmed the experimental reproducibility by performing two independent cDNA syntheses from the same RNA pool (Fig. 3b, see materials and methods for details). In addition, abundances measured previously for some bacterial and archaeal groups in the same environment using various quantitative real-time PCR or metagenomic methodologies were in agreement with this study [15]–[19]. One percent of the SSU and LSU ribo-tags were consistently not sorted into one of the three domains, instead being classified as “cellular organisms”. A closer inspection revealed that these represented short ribo-tags with comparably low taxonomic resolution power (not shown), rather than sequence tags of deeply rooting lineages or even of a “fourth domain of life”. In principle, though, this non-targeted approach has the potential to identify lineages, which are currently not seen by primer-based PCR methodology, like e.g. the Nanoarchaeota [27].

### Diversity and relative abundances of Bacteria and Archaea

**Figure 4:**
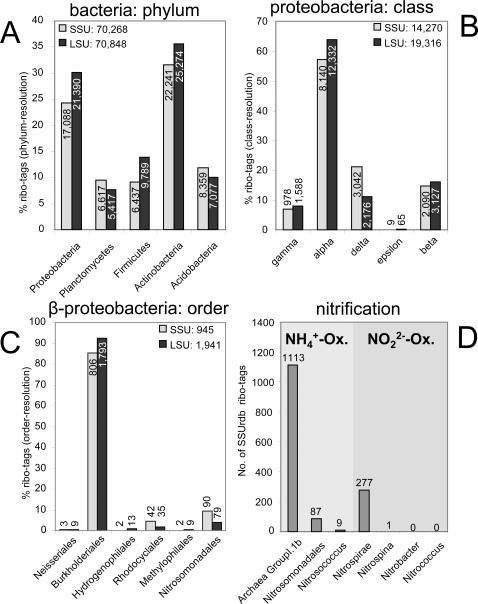
Relative distribution of bacterial SSU and LSU ribo-tags at different taxonomic resolutions and the abundance of prokaryotic groups involved in the nitrification process. a, fraction of phylum-resolution SSU and LSU ribo-tags affiliated to the five numerically dominant bacterial phyla (>5% of bacterial ribo-tags). Absolute numbers are additionally given. b, fraction of class-resolution SSU and LSU ribo-tags affiliated to the five proteobacterial classes. c, fraction of order-resolution SSU and LSU ribo-tags affiliated to the identified beta-proteobacterial orders. d, numbers of SSU ribo-tags from the seven archaeal and bacterial taxa involved in nitrification.

With 165,246 ribo-tags (85.5% of total ribo-tags), bacteria were by far the most abundant domain in the sample; they were also extraordinarily diverse. Nineteen out of 24 validly described bacterial phyla were identified (Fig. 2 and Table S6 in SI) in addition to 20 candidate divisions. The latter are recently discovered, deep-branching, poorly characterized groups. The dominant bacterial phyla in our sample, such as Actinobacteria (47,513 ribo-tags) and Proteobacteria (38,477 ribo-tags) are typically found in high numbers in soil microbiotas. While both databases showed similar results for the five most abundant phyla (Fig. 4a), incongruencies arose, where LSUrdb sequences were underrepresented (e.g. Chloroflexi, Verrucomicrobia, Bacteroidetes), or completely missing (e.g. Nitrospirae, Gemmatimonadetes, various candidate divisions, see Table S6 in SI). Even rare phyla, such as Chlorobi and Dictyoglomi (ca. 0.02% of all bacterial tags) were reliably detected by both rRNA databases. It is also noteworthy that the analysis remains congruent at a more detailed taxonomic resolution (class and order, Figures 4b and 4c) for the taxonomic groups, which are comparably well covered in both databases (e.g. Proteobacteria, Firmicutes, Actinobacteria). Approximately 2% (1803) of the bacterial SSU ribo-tags were distributed over 20 candidate divisions (Table S6 in SI). The numbers of ribo-tags ranged from one (WS2 and KSB1 candidate phylum) to several hundred (SPAM, OP8, OP10, NKB19, VC2), or in other words, from indications of the presence of a taxon, to a high confidence including a wealth of sequence information.

In-depth taxonomic profiling enables the analysis of microbial communities from various functional perspectives. We demonstrate this for the process of nitrification, the conversion of ammonia to nitrate via nitrite. Both SSU-based and LSU-based analyses consistently identified the Crenarchaeal candidate division GroupI.1b as the predominant archaeal taxon in the soil sample (Table S7 and Figure S5 in SI); this is consistent with earlier studies of the same habitat [16] [15]. Members of this group were recently identified as important players in ammonia oxidation [15], [20]. So it is perhaps not surprising that community-wide analysis of the different groups of bacteria and archaea harboring ammonia- and nitrite-oxidation capabilities (Fig. 4d) indicated that the groupI.1b Crenarchaeota were, at least from a numerical standpoint, the major ammonia-oxidizing group in our sample. The ribo-tag ratios between this group and ammonia-oxidizing bacteria were very similar to archaeal and bacterial amoA transcript ratios determined from the same cDNA preparation (12 vs. 16[15]). The subsequent nitrite-oxidation step appeared to be mainly performed by members of the Nitrospirae bacterial phylum. This result would have required seven independent quantitative real-time PCR assays to target the groups involved in nitrification when using traditional methods, whereas it represents only one of a variety of results obtained in this non-targeted approach. Additionally the respective functional groups are displayed in the context of the whole community.

As opposed to PCR-dependent approaches [10], [11], [26] that are confined to specific regions in the SSU rRNA molecule, our method allows the reassembly *in silico* of a full length “composite community” or consensus rRNA sequence for certain taxa. To illustrate this, the 1502 bp large SSU rRNA gene for groupI.1b of Crenarchaeota was assembled from 1105 ribo-tags. The resulting rRNA sequence differed by 3.1 % and 5.4 %, respectively, from the SSU rRNA sequences of the two fosmid clones, 29i4 and 54d9, isolated earlier from this habitat [17], [20].

### Diversity and relative abundances of Eukaryotes

**Figure 5:**
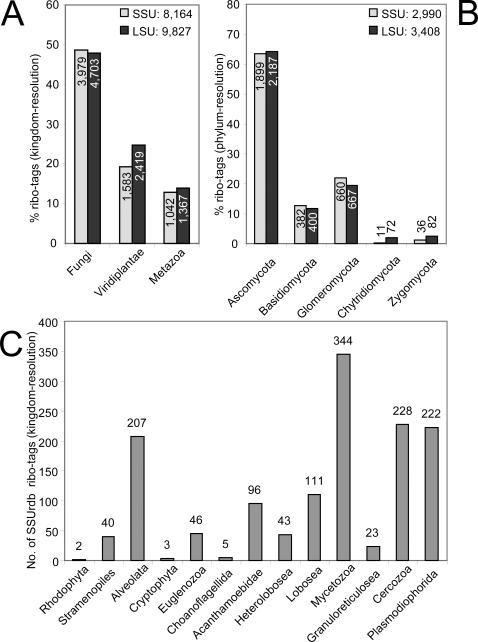
Relative and quantitative distribution of eukaryotic ribo-tags. a, fraction of kingdom-resolution SSU and LSU ribo-tags affiliated to the three numerically dominant eukaryotic kingdoms. b, fraction of phylum-resolution SSU and LSU ribo-tags affiliated to the five fungal phyla detected. The distribution was highly consistent in the SSUrdb and LSUrdb. c, numbers of SSU ribo-tags affiliated to the various protist kingdoms.

Although Eukaryotes are major players in soils and strongly influence the prokaryotic community structure, their diversity and abundance has received comparably little attention in molecular studies [28]. They account for ∼11% of the ribo-tags (i.e. cellular biomass) in the soil community. Due to in-depth sampling, we obtained 22,740 eukaryotic ribo-tags, with more than 80% of the tags having a taxonomic resolution at least to the kingdom level. The numerically dominant kingdoms were the Fungi, known as the major destruents in soil, as well as plants (Viridiplantae) and Metazoa (Fig. 5a), with ∼50%, ∼20% and ∼10% of the ribo-tags being consistently assigned by both reference databases. Within the Fungi, the phylum Ascomycota accounted for two thirds of the ribo-tags (Fig. 5b), followed by the phyla Glomeromycota and Basidiomycota.

As grazers, Protists play important roles in the soil food web by regulating microbial populations. Few LSU rRNA sequences are currently available for protists. Therefore, we estimated the Protist community composition using only the dataset of 1321 SSU ribo-tags (Fig. 5c). This is, to our knowledge, still the biggest molecular dataset of a protist community generated so far. It displayed the presence of slime molds (Mycetozoa) as the most abundant group, followed by Cercozoa, Plasmodiophorida and Alveolata. In addition, Lobosea, Acanthamoebidae, Heterolobosea, Euglenozoa and Stramenopiles were present with at least 40 ribo-tags in the sample.

The non-targeted and in-depth approach resulted in a dataset, which enabled a broad and holistic view onto a community and covered many of the trophic levels present. We are aware that the community profiles presented here are derived from the counting of ribosomal molecules, but not their genes, as is the case in DNA-based approaches. The ribosome content can vary considerably in the cells of an organism reflecting its physiological state; it also differs between taxa. Currently, few data are available; *E. coli* cells have been estimated to contain between 6,800 and 72,000 ribosomes, depending on the growth phase [29], unicellular eukaryotes likely have more ribosomes than microorganisms, and still higher is the ribosomal content of multi-cellular organisms like fungi, metazoans or plants. We consider the amount of ribo-tags as determined in this study to be a measure of a taxon's cellular biomass within a community [30], [31].

### Global functional analysis of putative mRNA-tags

**Figure 6:**
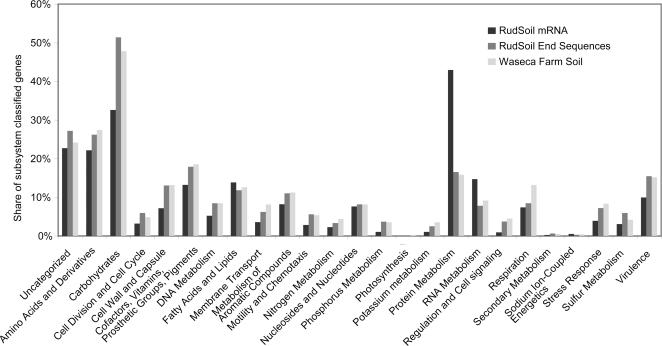
Global functional analysis of mRNA-tags, fosmid-derived end sequences from DNA of the same community [19] and shotgun-cloned DNA from a farm soil community [7]. All three datasets were subjected to automated analysis using the MG-RAST annotation procedure at the SEED (http://metagenomics.theseed.org). Percentages are expressed as the number of mRNA-tags assigned to a subsystem category, divided by the total number of mRNA-tags assigned to subsystems.

The 65,192 RNA-tags that did not give a significant hit against the rRNA reference databases were aligned against the Genbank non-redundant protein database. Homologues to 21,133 RNA-tags were found, showing that a considerable amount of assignable mRNA had been reversely transcribed (Table 1). We subjected those presumable mRNA-tags (2.1 Mbp) to a global functional analysis using the SEED database[32] and compared the meta-transcriptomic data to metagenomic data from (1) the same soil habitat (4.3 Mbp[19]) and (2) a different farm soil (145 Mbp[7]). Overall, the DNA-based (metagenomic) functional repertoire in both soils was surprisingly similar, as judged from the relative distribution of the functional subsystems (Figure 6). This indicates that a generally similar “pool of functions” is present in both soil communities, which consequentially indicates that functional investigations of soil communities based on DNA might always give similar global patterns. In contrast to this, categories involved in RNA and protein metabolism (transcription, translation, protein folding and degradation) were significantly over-represented in the meta-transcriptome compared to both metagenomes (2.7 to 4.0-fold, see Figure 6 and Figure S6 in SI), as one would expect to see for active organisms. Further differences between the transcriptome and the metagenomes were related to carbohydrate metabolism: while transcripts of proteins involved in the aerobic degradation of mono-, di- and oligo-saccharides and amino-sugars seemed to be less frequent than suggested by the metagenomes (by 2-fold or more; Figure S7 in SI), transcripts for fermentation, degradation of sugar alcohols and CO_2_-fixation were equally represented.

**Figure 7:**
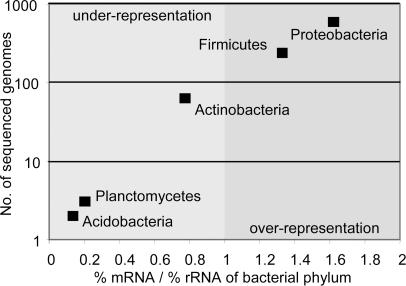
Logarithmic bi-variance plot of the number of publicly available genomes (as of September 2007) of the five numerically dominant bacterial phyla, as judged from the ribo-tags, versus the mRNA-tag based over- and under-representation, compared to the mean of SSU and LSU ribo-tag fraction. A ratio of one means that mRNA- and ribo-tags report the same fraction for the respective phylum.

Metagenomic analyses frequently assign protein-encoding genes to taxonomic groups by comparing them against the content of sequenced genomes to derive a taxonomic community profile [5], [6]. The simultaneously obtained rRNA and mRNA data provided us with the unique opportunity to validate these procedures. When we compared the ribo-tag profile with the community profile derived from taxonomic binning of the mRNA-tags using MEGAN, we observed a considerable shift for the five dominant bacterial phyla (Figure S8 in SI). These differences correlated strongly with the number of sequenced genomes of the different phyla (Fig. 7). This suggests that taxonomic binning solely based on protein encoding genes currently generates an artificial bias against groups with few sequenced genomes, and correspondingly over-represents phyla with many sequenced genomes. This problem, which is inherent to all metagenomic studies, will likely be overcome as more genome sequences of less represented phyla become available.

### Probing the metabolism of an uncultured low-abundant group

**Figure 8:**
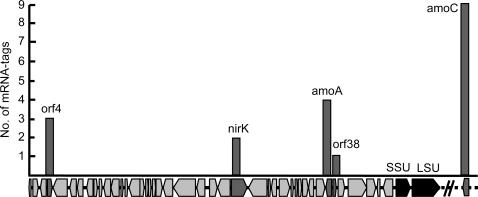
Abundance-dependent plot of presumably archaeal mRNA-tags onto the crenarchaeal fosmid clone 54d9 isolated from the same soil habitat (accession number: AJ627422). The x-axis represents the annotated open reading frames (orfs). Note that an *amoC* gene was not found on the fosmid and is therefore indicated as loosely affiliated.

1.7% of the mRNA-tags (ca. 36 kb) were identified as being of archaeal origin, similar to 1.5% of the ribo-tags (see Supplement Methods and Results S1 and Figure S5 in SI for details). These metatranscriptomic data allowed a first glimpse into the *in situ* activity of the yet uncultured soil Crenarchaeota from groupI.1b. Homologues to more than 80 mRNA-tags were functionally annotated in the databases. Besides transcripts of typical archaeal house-keeping gene products, those involved in ammonia oxdiation were predominant (Fig. 8); 13 mRNA-tags were derived from transcripts of the key metabolic enzyme ammonia monooxygenase (*amoA and amoC*). Furthermore, mRNA-tags of a putative copper-containing nitrite reductase (*nirk*) gene [20] indicated that this enzyme–as postulated for ammonia oxidizing bacteria–could be involved in the process of ammonia oxidation either under aerobic or anaerobic conditions[33]. These findings again hint for ammonia oxidation being the main energy metabolism in soil Crenarchaeota [34]. In addition, ten mRNA-tags could be related to the potential carbon metabolism. One mRNA-tag was derived from a homologue of methyl-malonyl-CoA mutase (MCM) and two from 4-hydroxybutyryl-CoA dehydratase (4-HBDH) homologues. These two gene products are, together with Acetyl-CoA/Propionyl-CoA carboxylase, diagnostic for a CO_2_ fixation pathway recently characterised in hyperthermophilic Crenarchaeota and suggested for marine crenarchaeota [35]. This indicates that a similar pathway of CO_2_ fixation might act in the soil crenarchaeota. Taken together, the metatranscriptomic data provide evidence for a chemolithoautotrophic lifestyle of this yet poorly characterised group.

With the non-targeted randomly primed reverse transcription approach, we can also assemble complete or nearly complete “composite” genes of specific lineages. A near full-length “composite” *amoC* transcript from the mRNA-tags of this analysis has been assembled (Figure S9 in SI). The deduced amino acid sequence covered 146 out of 189 positions (77%) of the archaeal sponge symbiont *Cenarchaeum symbiosum amoC* homolog [36] and had 88% identity to *C. symbiosum amoC*, similar to the 84% identity for *amoA* [15], [20].

### Conclusions

In conclusion, we have presented a rapid experimental and analytical approach that uses rRNA and mRNA to characterize microbial community structure and *in situ* function in-depth and simultaneously. This methodology will help (1) to identify microbial groups in complex communities; (2) to relate taxonomic groups to their ecological function (as demonstrated for the soil crenarchaeota); and (3) to efficiently monitor structural and functional community shifts caused by environmental changes. Furthermore, this approach enables for the first time the simultaneous quantitative assessment of the abundance of members of all three domains of life. The analytical power of this approach will continuously improve as sequence read lengths increase and as rRNA reference and genome databases continuously grow.

We believe that the analysis presented here could be used in parallel with mRNA enrichment procedures [13], [14] to ensure an efficient global analysis of the activity of naturally occurring assemblages, with one approach covering all trophic levels and domains as well as reasonable numbers of mRNA tags in one sequencing step (total RNA) and the other tool adding further in depth information from the enriched mRNA fraction.

## Materials and Methods

### Site description and soil sample processing

The soil samples were obtained from a sandy lawn in the environmentally protected area “Am Rotböll” (Germany, Hessen, 49°55′34″N 8°37′21.6″E, [15]). The soil is nutrient-poor with a pH of 7.06, and with water extractible organic nitrogen (WEON) and carbon (WEOC) of 0.9 and 7.6 mg kg dry weight soil^−1^, respectively. The top soil was sampled at a depth of 0–10 cm in January 2006 after a snow thawing period (soil temperature 5.5°C). Three replica within a distance of 1 m were withdrawn and kept in open plastic bags in the dark at 4°C for 40 hours. 100 g of the soil samples were sieved (2 mm mesh size) and equal amounts of the triplicates were subsequently mixed. Six grams of soil were then processed for RNA extraction.

### cDNA synthesis

Nucleic acid extractions (RNA and DNA) were performed using a modification of the method of [37], and as briefly described in [15]. 0.5 ml of both CTAB buffer and phenol:chloroform:isoamylalcohol (25:24:1, pH 6.8) were added to a lysing matrix E tube (Q-Biogene) containing 0.5 g of soil. The cells were lysed in a FastPrep machine (Q-Biogene) at speed 5.5 for 30 seconds, followed by nucleic acid precipitation with PEG 8000. Total nucleic acids were subjected to Dnase treatment and the remaining total RNA was used as template for random hexamer-primed reverse transcription performed independently at 37°C and 50°C, respectively. The successful and complete hydrolysis of DNA was probed by archaeal and bacterial *amoA* specific quantitative real time PCR, which detected specific products in the cDNA samples after reverse transcription but not in parallel treated RNA samples, where no cDNA synthesis had taken place ([15], data not shown). The subsequently generated double-stranded (ds) cDNA ranged from approximately 100 to 1500 bp in length (not shown). Approximately one microgram of ds cDNA was generated.

### Pyrosequencing

Sequencing was performed as described previously [15]. The 37°C and 50°C samples were kept separate during pre-sequencing processing. Independent test-sequencing was performed on a Roche GS20 sequencer (Roche Applied Sciences/454 Life Sciences, Branford, CT) for both samples, producing small subsets of RNA-tags (37°C = 4141 and 50°C: n = 1985). Both samples were subsequently pooled for one full run resulting in 314,041 sequences [15]. Re-analysis of the sequences resulted in 258,411 high-quality RNA-tags.

### Reference Databases

Two rRNA reference sequence databases were constructed for community structure analysis using MEGAN [23]. The sequences therein were linked to taxa in the NCBI Taxonomy (as of June 2007), extended with 28 archaeal candidate divisions [38] (see Tables S1 and S2 in SI). The Small- and Large Subunit rRNA Reference Databases (hereafter SSUrdb and LSUrdb, respectively) were constructed by combining sequences from several public databases. The SSUrdb includes sequences from the RDP-II release 9.39 [22] (bacteria), the SILVA SSURef database release 89 [21] (eukaryotes and archaea), and sequences from the dataset described in [38] (archaea). The LSUrdb includes sequences from the Silva LSURef database release 90 [21]. Bacterial SSU rRNA sequences were retrieved from RDP-II [22]. More than 112,000 high quality sequences from isolates and uncultured strains were downloaded as FASTA files. The selection was made according to their taxonomic affiliation in the NCBI taxonomy. Sequences with low taxonomic resolution (e.g. “unclassified bacteria”, “environmental samples”) were mostly omitted until the “order” taxonomic level. Only in phyla with comparably few reference sequences and/or a relatively poorly refined taxonomy (e.g. the phyla Acidobacteria and Verrucomicrobia, and various candidate divisions), those sequences were included. All sequences were screened for vector contamination using the *cross_match* program from the Phred/Phrap package [39] against NCBI's UniVec database. All identified vector subsequences were removed from the database sequences. In some RDPII derived sequences, the entry contained more than the SSU rRNA gene, i.e. flanking regions and the LSU rRNA gene. In order to remove those, all sequences longer than 1,550 bp were aligned to the remainder of the sequences in the database (those within the expected length of an SSU rDNA gene), using BLASTN. Based on these alignments, any regions without significant similarity (E≤1e-6) were cropped, leaving only the SSU rRNA gene.

All eukaryotal and most archaeal SSU rRNA sequences were retrieved from the SILVA project [21]. The SSURef database version 89 was retrieved as an ARB file. 24,197 aligned eukaryotal sequences including 331 mitochondrial and 641 plastid sequences were analyzed and chosen for our database based on a correct taxonomic affiliation in the NCBI taxonomy and a comparatively high taxonomic resolution using the software package ARB [40]. Sequences were exported as FASTA files using a filter (including positions between 1000 and 43284 of the ARB alignment) for eliminating sequence information not belonging to the SSU rRNA gene.

The archaeal part of SSUrdb reference database contained 944 sequences from cultured strains, extracted from the SILVA SSURef database, again based on a correct and high resolution taxonomic affiliation in the NCBI taxonomy. Many archaeal lineages today consist exclusively of uncultured representatives. Those are not well resolved in the NCBI taxonomy, but deposited as “uncultured archaea” or “environmental samples”, which prevents an informative taxonomic grouping of sequences belonging to those lineages. To overcome this, we have extended the NCBI taxonomy with 28 archaeal candidate divisions as described in [38] and exchanged the taxonomic affiliation of the effected sequences in the SSUrdb accordingly. Altogether 544 sequences are distributed over those groups (extracted from the ARB dataset used in [38]; see Table S2 in SI). The archaeal part therefore consists of 1,490 sequences and the whole SSUrdb of 135,160 sequences in total.

The large subunit reference database (LSUrdb) was generated from the SILVA database project. The LSURef database version 90 was retrieved as ARB file. The sequences therein were analyzed and chosen based on a correct taxonomic affiliation in the NCBI taxonomy reflected in the sequence alignment and a comparatively high taxonomic resolution using ARB. Sequences were exported as FASTA files using a filter (including positions between 66155 and 129011 of the ARB alignment) for eliminating sequence information not belonging to the LSU rRNA gene. Vector sequences were removed as described above. The LSUrdb consists of 6,247 sequences, of which 2,759 belong to Bacteria, 130 to Archaea (107 cultured and 23 uncultured) and 3,358 Eukaryotes (including 122 mitochondrial and 491 plastid sequences).

As MEGAN utilizes the FASTA header in the BLAST output to identify the corresponding taxon (see below), the headers of all files in the reference databases contain the Genbank, RefSeq or EMBL accession number as identifier, to enable taxon identification with MEGAN (see below).

### Taxonomic assignment of RNA-tags using MEGAN

All RNA-tags in the sample were compared to both of the rRNA reference databases using the NCBI *blastall* implementation of BLASTN (default parameters, except setting the maximum number of hits to 100). Analysis of BLAST output files was performed using the MEGAN software version 1beta18 [23]. This software reads the results of a BLAST comparison as input and attempts to place each read on a node in the NCBI taxonomy. This is performed by the LCA algorithm that assigns each RNA-tag to the lowest common ancestor in the taxonomy from a subset of the best scoring matches in the BLAST result (absolute cutoff: BLAST bitscore 86, relative cutoff: 10% of the top hit). RNA-tags that have no BLAST matches are assigned to the special node “no hits” and those unassigned due to algorithmic reasons (e.g. below an applied threshold) are placed on the special node “unassigned”. The result of the analysis is displayed as a tree representation of the NCBI taxonomy (as of June 2007). To enable identification of the species involved in the BLAST hits, we employed lookup-tables that map GI accessions to taxon IDs obtained from the NCBI website.

### Database refinement, analysis pipeline adjustment and testing

In order to evaluate the performance and stability of our taxonomic binning method using MEGAN and the generated reference databases, we used LSU and SSU rRNA test sets containing simulated ribo-tags. Those consisted of 100 bp long sequence fragments and were obtained by randomly cropping out a 100 bp window from a given rRNA sequence. This procedure was repeated 200 times resulting in a statistical coverage of 13.3 for each position in SSU rRNA (for an assumed length of 1500 bp) and 6.7 for LSU rRNA sequences (for an assumed length of 3000 bp) within the test set. Datasets were iteratively compared by BLASTN against the reference database(s) and analyzed using MEGAN.

Simulated SSU ribo-tag datasets were aligned to the LSUrdb and vice versa, to estimate the potential of “cross-contamination”, i.e. the assignment of SSU rRNA-derived ribo-tags as LSU rRNA ribo-tags and vice versa. No such assignments were observed above a BLASTN score of 71 bits. Manual inspections of the alignments from the simulated data lead us to choose 86 bits as the minimum BLASTN score for an RNA-tag to be considered as ribo-tag. These alignments are always longer than 40 bases and correspond to an e-value of 2e-16 for SSUrdb alignments. In addition, a relative threshold was introduced in the taxonomic binning procedure applying MEGAŃs LCA algorithm. Here, ten percent was chosen as cut-off, which means that the taxonomic information of all reference sequences on the BLAST hit list which are within 10% of the score of the BLAST top hit were included in the taxonomic binning of the respective ribo-tag. Increasing this cutoff to 20% or more, i.e. making the taxonomic assignment of a ribo-tag less strict, led to a very minor shift in taxonomic resolution (data not shown), showing that the chosen relative cut-off is already rather relaxed.

The rRNA reference databases were screened for taxonomically wrongly assigned reference sequences using simulated ribo-tag datasets. Since those consisted of ribo-tags of known origin, an assignment congruent with the taxonomic resolution power of the database for the respective taxon was expected. Where results deviated from these expectations, they were manually inspected. Especially the BLAST hit lists of ribo-tags with poor taxonomic resolution (mainly the taxonomic levels “cellular organism”, “domain” and several prokaryotic “phyla”) were analysed in detail. For example, a single acidobacterial reference sequence wrongly affiliated to another bacterial phylum would result in a drastic under-representation of acidobacteria, because the LCA algorithm would group the acidobacterial ribo-tags at the bacterial domain level. In the analysis of a natural community, this would introduce a strong artificial community shift, biasing against acidobacteria. Suspicious reference sequences were compared by BLAST against NCBI non-redundant nucleotide database and removed from the reference database. The test set was subsequently compared to the refined reference database, to verify an improved performance for the respective taxon and/or to identify additional wrongly affiliated reference sequences. Applying this iterative procedure, more than 600 bacterial sequences with wrong affiliations in the NCBI taxonomy were removed from the SSUrdb.

In order to extend the sensitivity and robustness analysis (see Supplement Methods and Results S1 in SI), we attempted to simulate the situation where ribo-tags would be as similar to the database as in median case, by removing entries from the database. Database filtering was carried out by aligning the full-length SSU and LSU rRNA sequences from seven selected test species to all entries in the respective reference database, using BLASTN with modified scoring parameters (mismatch penalty = 1, gap open cost = 2, gap extension cost = 1). In all cases where the full-length test sequence showed higher similarity than the threshold to a database sequence, this sequence was removed from the database. The procedure was carried out independently for each test species with the respective sample median similarities used as thresholds (98% for the SSUrdb and 93% for the LSUrdb), thus producing 7 filtered versions of the LSUrdb and SSUrdb. For the SSUrdb, the lowest decile similarity (86%) was also removed in a separate filtering. The simulated SSU and LSU ribo-tags from the test species were then aligned to respective filtered database versions using BLASTN and taxonomically assigned using MEGAN, as described above.

### Assembly of a “composite community” rRNA sequence

Using MEGAN, all 1,113 putative SSU ribo-tags assigned to the archaeal GroupI.1b were extracted from the dataset. The assembly program CAP3 [41] was then used to attempt to assemble the data (default parameters) which resulted in a single contiguous sequence contig with a length of 1,502 bp assembled from 1105 of the extracted ribo-tags.

### Functional analysis of putative mRNA-tags

All RNA-tags without a significant similarity against the rRNA reference databases (BLAST score threshold below 86 bits), were translated in all six reading frames and aligned to the NCBI non-redundant protein database (release of June 25, 2007) using BLASTX [42] and analysed with MEGAN. The resulting tags with at least one BLAST alignment producing a bitscore of 30 or above were assigned as putative mRNA transcripts. Manual inspection revealed that sequences with BLAST hits close to a bitscore of 30 produce apparently meaningful alignments. In addition, the coverage of the ribosomal reference databases, compared to the true distribution of all existing ribosomal sequences, is expected to be much better than the coverage of Genbank nr compared to all existing protein coding sequences. This assumption, together with the short lengths of the translated reads (approximately 30 amino acids) means that possibly true mRNA transcripts would be missed, were we to use a higher bitscore limit.

Putative mRNA sequences were annotated using the MG-RAST (Meta Genome Rapid Annotation using Subsystem Technology; v1.2) server at the Argonne National Library (http://metagenomics.nmpdr.org), using subsystem-based annotation based on the SEED database [32]. Subsystems are groups of genes, or functional roles, acting together in a biological process, e.g. in a metabolic pathway. These are grouped into subsystem categories. The MG-RAST annotation pipeline assign some putative genes to more than one subsystem, i e predicts that these genes have multiple functions. In such cases, each assignment was counted in the statistic Total Subsystem Assignments in Table S8 in SI (so that the gene was counted more than once). As a consequence of this, the sum of the relative subsystem counts (“Share of subsystem classified genes”, Figures 6, Figures S6 and S7 in SI) add up to more than 100%. In addition to the putative mRNA-tags, subsystem annotation of two metagenome datasets was also carried out using the same methodology. The first of these contains genomic sequences from a sample collected previously from the same site[19] and the second from farm soil [7] (see for details Table S8 in SI). For each subsystem, the relative population was calculated, i.e. the putative genes assigned to a particular subsystem divided by the total number of genes assigned to subsystems. In addition to manual comparison of relative populations between the samples putative mRNA-tag dataset and genomic sequence from the same site, these datasets were compared statistically using the method described in[43] (Table S9 in SI). In order to rule out that the difference in abundance was not an artifact caused by the shorter length of the putative mRNA-tags, compared to the longer genomic reads obtained using Sanger sequencing, the genomic reads from [19] were fragmented to a number of shorter reads and re-analysed using the MG-RAST server. From each genomic read, seven fragments were randomly generated with a length identical to the average length of the mRNA-tags (98bp), such that the total sequence length was close to that of the un-fragmented sample. The distribution of subsystem categories for the short fragments did not show any deviances larger that 10% compared to the original dataset, for the subsystems or subsystem categories abundant in the mRNA sample (data not shown). This showed that the observed abundance is not an artifact caused by shorter read length of the mRNA-tags. A study where 454 transcripts were functionally categorized and compared to Sanger sequenced ESTs in the plant *Medicago*, also showed that 100bp length is sufficient for meaningful functional classification comparable to traditional ESTs [44].

The putative mRNA-tags were taxonomically binned by analysing the output file of the BLASTX comparison against the NCBI nr protein database with MEGAN. The same parameters as described above for the ribo-tag analysis were applied (except a minimum score of 30 bits). The resulting taxonomic community profile was manually compared to that from the corresponding ribo-tag analyses. In addition, higher score cutoffs (40 and 50 bit) were applied on the mRNA-tags dataset, which gave essentially the same community profile (not shown).

Nine mRNA-tags which were found to have similarity to the ammonia monooxygenase subunit C gene (*amoC*) of archaea, were translated to protein sequence using the reading frames corresponding to the BLASTX hits, and aligned to the *Cenarchaeum symbiosum* AmoC protein sequence using CLUSTAL X [45].

### Data deposition

The SSUrdb and LSUrdb are made available for download as compressed fasta files from http://www.bioinfo.no/services/community-profiling. The sequences reported in this paper have been submitted to GenBank, under accession number SRA001014.

## Supporting Information

Methods and Results S1 This file contains supplementary methods and results
(0.08 MB DOC)

Figure S1 Distribution of sequence difference between assigned SSU ribo-tags and their top scoring BLAST match in the SSUrdb. Similarity is defined as the number of nucleotide identities in the BLASTN alignment divided by the total length of the ribo-tag.
(0.17 MB TIF)

Figure S2 Distribution of sequence difference between assigned LSU ribo-tags and their top scoring BLAST match in the LSUrdb. Similarity is defined as the number of nucleotide identities in the BLASTN alignment divided by the total length of the ribo-tag.
(0.20 MB TIF)

Figure S3 3D Bar plot showing the number of correctly assigned simulated SSU ribo-tags at different taxonomical levels. 200 ribo-tags of length 100 bp were randomly simulated from seven test species and compared to a modified version of the SSUrdb, filtered in order to exclude all sequences more than 98% similar to the species test sequence (the median similarity of the SSU ribotags in the sample). Note that no order level in Crenarchaeote 54d9 is defined.
(0.92 MB TIF)

Figure S4 3D Bar plot showing the number of correctly assigned simulated LSU ribo-tags at different taxonomical levels. 200 ribo-tags of length 100 bp were randomly simulated from seven test species and compared to a modified version of the LSUrdb, filtered in order to exclude all sequences more than 93% similar to the species test sequence (the median similarity of the SSU ribotags in the soil sample to reference sequences in the LSUrdb). Note that no order level in the Crenarchaeote 54d9 is defined.
(0.74 MB TIF)

Figure S5 MEGAN comparison of archaeal rRNA based and mRNA based community profile. The taxonomic affiliation of an RNA-tag is based on the Blast hits within 10% of the top Blast Bit score.
(0.94 MB TIF)

Figure S6 Functional analysis of DNA, protein and RNA metabolism subsystems in Rudsoil mRNA-tags, fosmid-derived end sequences from DNA of the same community (Treusch et al., 2004) and shotgun-cloned DNA from a farm soil community (Tringe et al., 2004). All three datasets were subjected to automated analysis using the MG-RAST annotation procedure at the SEED (http://metagenomics.theseed.org).
(0.26 MB TIF)

Figure S7 Functional analysis of carbohydrate metabolism subsystems in Rudsoil mRNA-tags, fosmid-derived end sequences from DNA of the same community (Treusch, 2004) and shotgun-cloned DNA from a farm soil community (Tringe, 2004). All three datasets were subjected to automated analysis using the MG-RAST annotation procedure at the SEED (http://metagenomics.theseed.org).
(0.20 MB TIF)

Figure S8 Fraction of phylum-resolution SSU and LSU ribo-tags (in %) affiliated to the five numerically dominant bacterial phyla (>5% of bacterial ribo-tags) compared to mRNA-tag derived fraction for each phylum.
(0.15 MB TIF)

Figure S9 The soil crenarchaeota “composite” AmoC protein. Alignment of 9 amoC mRNA-tags (translated into amino acid sequence) against the AmoC protein of Cenarchaeum symbiosum (DQ397580), a member of the marine groupI.1a Crenarchaeota. Mismatches between the sequences are shaded gray. The nine mRNA-tags form three fragments which cover 77% of the C. symbiosum AmoC with 88% sequence identity.
(1.78 MB TIF)

Table S1 Schematic overview of the sequence content of SSUrdb and LSUrdb.
(0.03 MB DOC)

Table S2 Archaeal candidate divisions implemented into the NCBI taxonomy.
(0.05 MB DOC)

Table S3 Result of BLASTN of SSU rRNA test dataset derived from 43 species against SSUrdb.
(0.12 MB DOC)

Table S4 Result of BLASTN of LSU rRNA test dataset derived from 43 species against LSUrdb.
(0.12 MB DOC)

Table S5 Sensitivity analysis results for the SSUrdb and LSUrdb.
(0.14 MB DOC)

Table S6 The bacterial community structure in soil.
(0.11 MB DOC)

Table S7 The archaeal community structure in soil.
(0.04 MB DOC)

Table S8 Statistics from functional analysis of putative mRNA-tags.
(0.03 MB DOC)

Table S9 Functional subsystems with significantly higher abundance in putative mRNA-tags compared to a metagenomic sample.
(0.03 MB DOC)
