# Supplementary material for: Simultaneous Assessment of Soil Microbial Community Structure and Function through Analysis of the Meta-Transcriptome
Source: PLoS One. 2008 Jun 25;3(6):e2527. doi: 10.1371/journal.pone.0002527 (PMC2424134; doi:10.1371/journal.pone.0002527)
Supplement: Methods and Results S1 — This file contains supplementary methods and results (0.08 MB DOC) [file pone.0002527.s001.doc]

SUPPLEMENTARY METHODS AND RESULTS

**Sensitivity and robustness analysis of the taxonomic binning approach**

In order to test the sensitivity and robustness of our taxonomic binning approach with MEGAN and the LSUrdb and SSUrdb, we generated an SSU and an LSU test set of simulated ribo-tags. They were prepared from the full-length small- or large subunit rRNA sequence from 43 selected test species, composed of 32 bacterial, five archaeal and six eukaryotic representatives (see Materials and Methods for details).

SSUrdb (see supplement Table S3 in SI for details): On the domain level, 100% of the archaeal and eukaryal ribo-tags and 99.8% of bacteria-derived ribo-tags were correctly identified. Ten ribo-tags, which were only identified as "cellular organisms" were from cyanobacteria. Here, the close similarity to plastid sequences from eukaryotes resulted in a decrease in resolution. The resolution power of all archaeal and eukaryal ribo-tags and of 97% bacterial ribo-tags was higher than the domain level. More than 90% of bacterial and archaeal ribo-tags were assigned to the correct phylum, and in 22 out of 26 classes at least 80% of the ribo-tags were correctly grouped, highlighting also that the newly implemented archaeal taxonomy gives reliable results. Similarly, in 21 out of 26 orders, more than 70% of the simulated ribo-tags were correctly affiliated. In the 5 remaining orders, the lower relative representation was due to the usage of taxonomically less well defined sequences in the SSUrdb. The reason for this was a trade-off between providing sufficient reference sequence space and the taxonomic resolution of the reference sequences (e.g. for the acidobacteria). These results show that (1) the community composition can be robustly measured on the domain level, but also with a good resolution until the order level. (2) A comparably small artificial community shift is introduced by the database at the order level.

LSUrdb (see supplement Table S4 in SI for details): The LSUrdb differs in size and sequence composition from the SSUrdb (see supplement Table S1 in SI for details). Primarily, it should be regarded as a suitable internal control for the the SSUrdb in its present state. Founding the community analysis on the two most commonly used marker molecules is a major advantage compared to purely SSU rRNA based studies. Our test dataset indicates that the LSUrdb had a similar taxonomic resolution as the SSUrdb; all eukaryotal and archaeal and 99.7% bacterial ribo-tags were correctly assigned, again without any incorrectly assigned ribo-tags. All microbial orders were represented with at least 70% of the respective sequences. The LSUrdb corroborates the results obtained with the SSUrdb, although with a restriction of considerably reduced sequence space (e.g. for many of the bacterial candidate divisions only SSU rRNA sequences are currently available). ­ Overall, these tests on SSUrdb and LSUrdb did not result in any incorrect taxonomic assignment of a ribo-tag.

The tests described above used simulated ribo-tags with sequences identical to those in the reference databases. While this provides a good indication of the resolution and sensitivity of the taxonomic binning approach for sample ribo-tags essentially identical to the reference sequences (i.e. originating from organisms with known rRNA sequences), it is probable that a fraction of the ribo-tags in an environmental sample will differ significantly from reference sequences. To measure this difference, we analyzed the similarity of ribo-tags from the soil sample to their closest reference sequence. This was carried out separately for the LSU and SSU ribo-tags. Similarity was defined as the number of identical nucleotides in the BLASTN alignment divided by the length of the ribo-tag. The distributions of sequence difference are shown in Figures S1 and S2 in SI. The median difference (50% of the ribo-tags) was 2% for SSUrdb and 7% for LSUrdb. Difference for the lowest decile (ten percent quantile) was 14% for SSUrdb and 31% for LSUrdb. Differences appeared to be approximately exponentially distributed.

In order to extend the sensitivity and robustness analysis, we simulated the situation where ribo-tags would be as similar to the database as in median cases for seven of the test species. This was done by removing reference sequences with ≥ 2% (SSUrdb) and ≥ 7% (LSUrdb) similarity to a test sequence from the database prior to the taxonomic assignment of simulated ribo-tags (see Materials and Methods). In addition, all reference sequences with ≥ 86% similarity to test sequences were removed from the SSUrdb, to simulate a situation similar to the lowest decile similarity, as determined above. Results are shown in Table S5 and Figures S3 and S4 in SI.

No significant decrease in taxonomic resolution followed by filtering the SSUrdb at the median similarity level in most cases. At the class level, more than 71% of the simulated ribo-tags were correctly assigned for all seven test species and over 95% for four of them (Table S5 and Figure S3 in SI). At the domain level, all of the simulated ribo-tags were correctly assigned. Overall, no ribo-tag was incorrectly assigned, again showing the robustness of our taxonomic binning approach with the SSUrdb. We calculated the probability for not observing no false assignments (out of 800) at the order level, using an exact binomial test with a true false discovery rate of 0.5% or more (p=0.01813). Thus, our simulation indicates that the false discovery rate is lower than this at median similarity. These predictions are obviously only valid given that our relatively small test set contains organisms representative of the database as a whole, in terms of “surrounding sequence space”, or in other words the density of known sequences in the database compared to the sample.

When removing reference sequences ≥ 86% similar (at the lower decile level; see Table S5 in SI), assignments were significantly biased between test species at all taxonomical levels. Nonetheless, as many as 50% of the ribo-tags for all test species could be correctly assigned to phylum level or better. Only 5% of the total ribo-tags were incorrectly assigned and 18% remained unassigned (i.e. not classified as SSU rRNA). This indicates that our binning approach generates a comparably low number of false positives. However, this simulation approach is not sufficiently extensive to estimate the false discovery rate for sequences from organisms far from known sequence space. Interestingly, there seems to be no correlation in our test set between the number of unassigned ribo-tags from a test species to the number incorrect assignments.

In the simulated LSU ribo-tags, the resolution and sensitivity decreased significantly when filtering the reference database from sequences ≥ 93% similar (at the median level; see Table S5 in SI). This is not surprising giving the smaller size of the database and the small number of reference sequences for some phyla. For five of the seven test species, however, more than 70% of the ribo-tags were still correctly assigned at least to the class level. In contrast, sequences from the two phyla Spirochaetes and Aquifacae were almost entirely filtered from the reference database with this cutoff, causing a drastic loss of taxonomic resolution and the "disappearance" of the entire phylum Aquifacae. Remarkably, only a small proportion of the *A. aeolicus* ribo-tags was incorrectly assingned to a different phylum, but the majority remained unclassified This shows that for most phyla, results based on LSU ribo-tags appear relatively unbiased, whereas some may introduce a significant bias. In total, five percent of the simulated LSU ribo-tags were incorrectly assigned and 24% remained unassigned. This is comparable to the SSUrdb perfomance at the lower decile level.

Given the current number of sequences in the LSUrdb, and ribo-tag read length of around 100bp, the LSUrdb is mainly regarded as an internal control for results obtained with the SSUrdb.

Out of the SSU ribo-tags in the soil sample, 955 (1%) had only one BLAST match above the applied bitscore cutoff. The corresponding number for LSU ribo-tags was 6,724 (7%). In these cases, assignment will only be based on the best match and thus expected to be less robust. This difference in robustness between the reference databases is comparable to false assignments at the median level in the simulations described.

In summary, the combined, independent usage of both reference databases on a dataset appears to provide a robust and relatively unbiased analysis of the taxonomic composition of a community, especially for SSU ribo-tags. The false discovery rate is expected to be lower than 0.5% for SSU ribo-tags and around 5% for LSU tags. As the ribo-tag length is limited by the pyrosequencing technology used (currently 250 bp are already feasible, with potential for up to 400bp in the near future), we simulated the performance of the taxonomic binning with 200bp long ribo-tags. Especially the SSUrdb performs considerably better with 200 bp at higher taxonomic resolution (data not shown), which will make the taxonomic community profile even more reliable.

**Taxonomic binning of archaeal mRNA-tags**

The metabolism of groupI.1b Crenarchaeota from soil is, due to the lack of cultured representatives largely unknown. We have taxonomically binned the putative mRNA-tags using MEGAN, applying the same relative threshold as with the ribo-tag analysis on a BLASTX comparison against the NCBI nr protein database (BLAST hits within 10% of the top-hit were included in the binning procedure). 237 mRNA-tags were identified as of archaeal origin. This accounts for 1.1% of all mRNA-tags, which is similar to the proportion of archaeal ribo-tags (1.5%). Remarkably, 2/3 of the mRNA-tags were affiliated with lineages within the Euryarchaeota and only 1/3 with Crenarchaeota, which is in strong contrast to the SSU and LSU ribo-tag derived community profile (Figure S3 in SI). The root cause for this discrepancy is likely the genetic information deposited in the NCBI nr database. There is very limited information about the genomic repertoire of groupI.1b Crenarchaeota, as no genome is sequenced, and only few genomic fragments have been deposited in the databases, obtained through metagenomic studies [1-3]. This probably leads to the taxonomic binning of mRNA-tags to other archaeal groups. Based on the ribo-tag analysis from the same experiment, where the crenarchaeal groupI.1b consistently accounts for more than 98% of the LSU and SSU ribo-tags (Table S6 in SI), we assumed that most of the archaeal mRNA-tags are indeed derived from this group and not from euryarchaeal or other crenarchaeal lineages. Including only the BLAST top hit into the taxonomic binning increased the number of putative archaeal mRNA-tags to 360. Although most of the identified homologues encoded hypothetical proteins, approximately 80 homologues were functionally annotated to a more or less extend.

1. Quaiser A, Ochsenreiter T, Klenk HP, Kletzin A, Treusch AH, et al. (2002) First insight into the genome of an uncultivated crenarchaeote from soil. Environ Microbiol 4: 603-611.

2. Treusch AH, Kletzin A, Raddatz G, Ochsenreiter T, Quaiser A, et al. (2004) Characterization of large-insert DNA libraries from soil for environmental genomic studies of Archaea. Environ Microbiol 6: 970-980.

3. Treusch AH, Leininger S, Kletzin A, Schuster SC, Klenk HP, et al. (2005) Novel genes for nitrite reductase and Amo-related proteins indicate a role of uncultivated mesophilic crenarchaeota in nitrogen cycling. Environ Microbiol 7: 1985-1995.
