# Supplementary figures and images for: Simultaneous Assessment of Soil Microbial Community Structure and Function through Analysis of the Meta-Transcriptome
Source: PLoS One. 2008 Jun 25;3(6):e2527. doi: 10.1371/journal.pone.0002527 (PMC2424134; doi:10.1371/journal.pone.0002527)

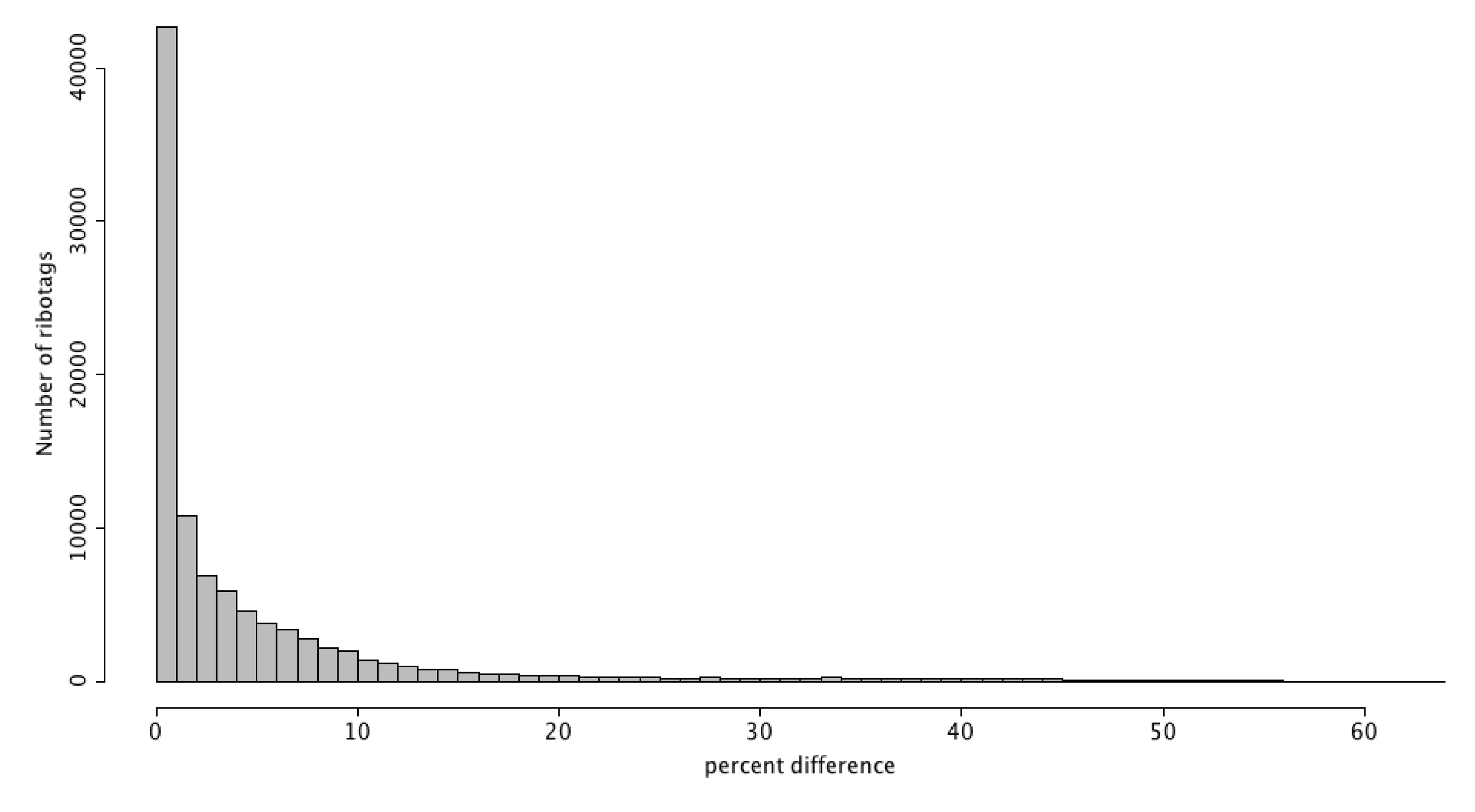

Supplement: Figure S1 — Distribution of sequence difference between assigned SSU ribo-tags and their top scoring BLAST match in the SSUrdb. Similarity is defined as the number of nucleotide identities in the BLASTN alignment divided by the total length of the ribo-tag. (0.17 MB TIF) [file pone.0002527.s002.tif]

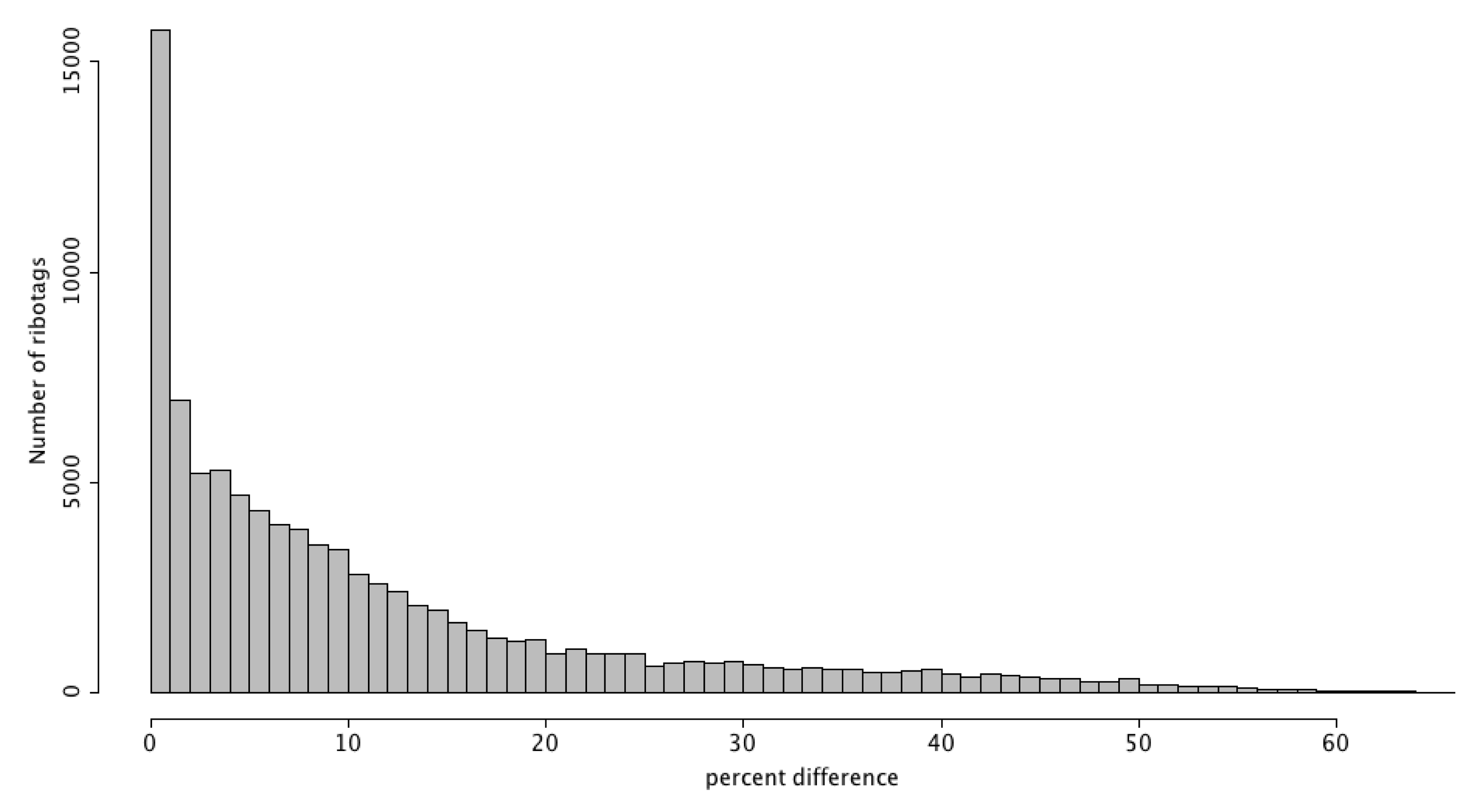

Supplement: Figure S2 — Distribution of sequence difference between assigned LSU ribo-tags and their top scoring BLAST match in the LSUrdb. Similarity is defined as the number of nucleotide identities in the BLASTN alignment divided by the total length of the ribo-tag. (0.20 MB TIF) [file pone.0002527.s003.tif]

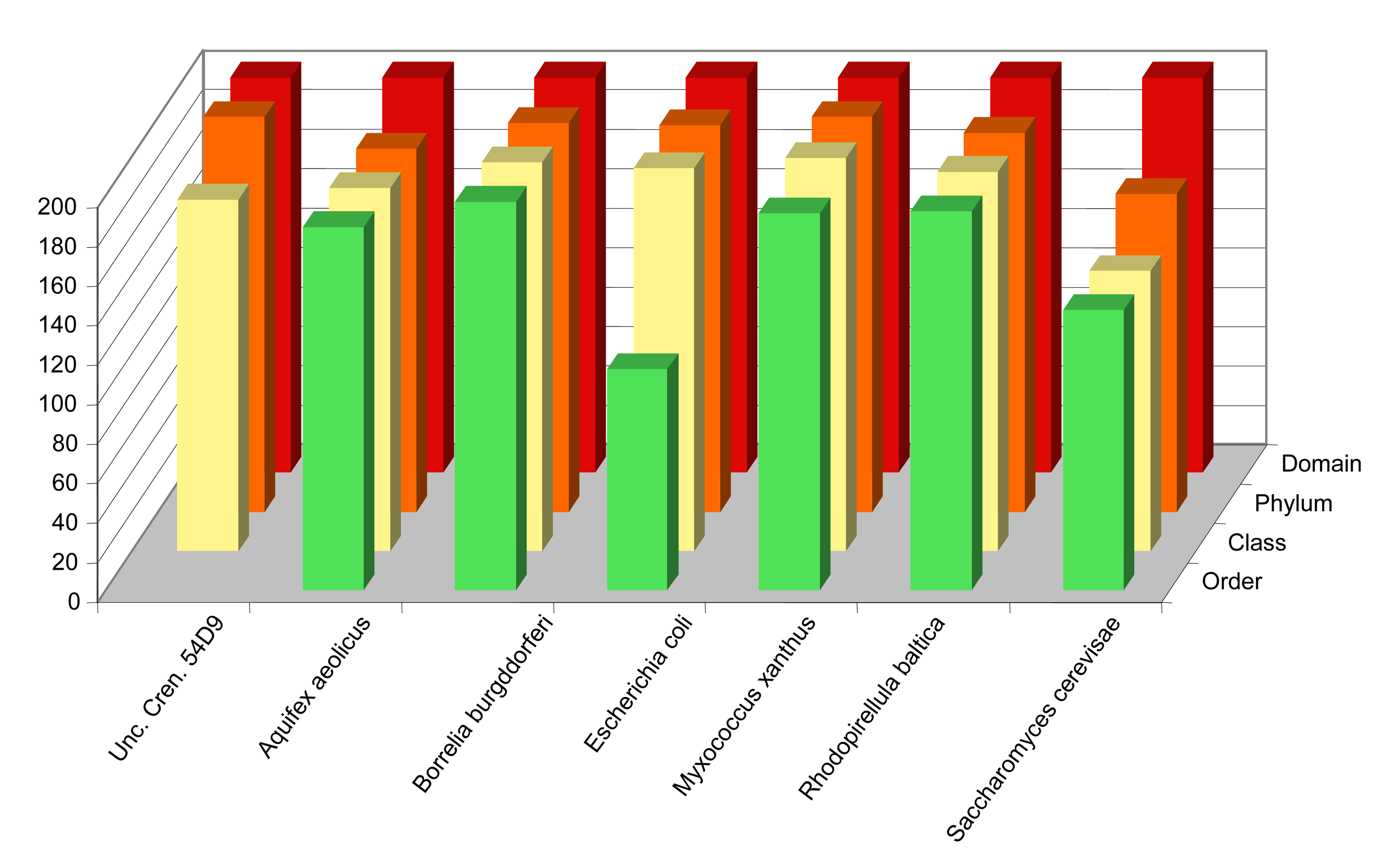

Supplement: Figure S3 — 3D Bar plot showing the number of correctly assigned simulated SSU ribo-tags at different taxonomical levels. 200 ribo-tags of length 100 bp were randomly simulated from seven test species and compared to a modified version of the SSUrdb, filtered in order to exclude all sequences more than 98% similar to the species test sequence (the median similarity of the SSU ribotags in the sample). Note that no order level in Crenarchaeote 54d9 is defined. (0.92 MB TIF) [file pone.0002527.s004.tif]

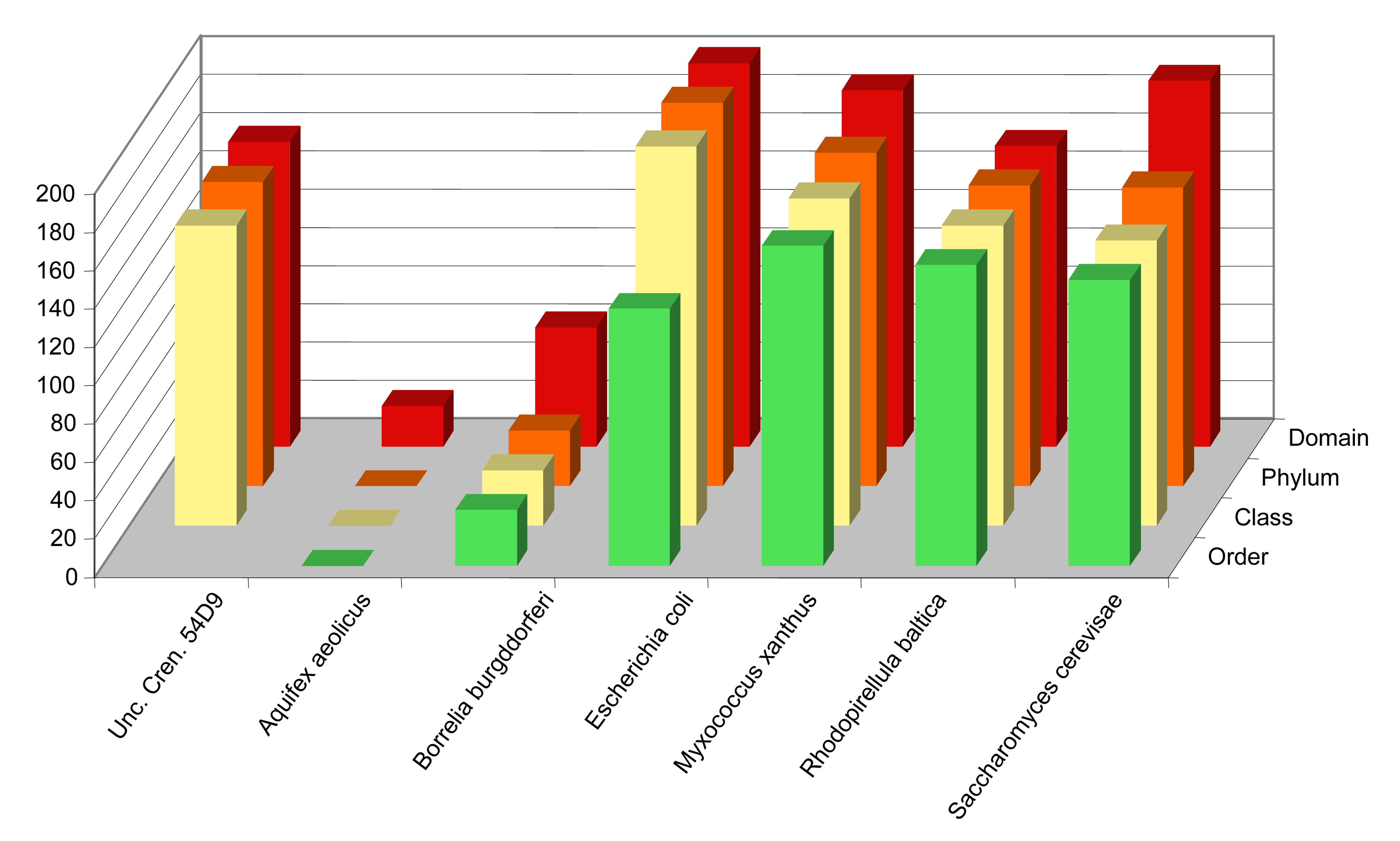

Supplement: Figure S4 — 3D Bar plot showing the number of correctly assigned simulated LSU ribo-tags at different taxonomical levels. 200 ribo-tags of length 100 bp were randomly simulated from seven test species and compared to a modified version of the LSUrdb, filtered in order to exclude all sequences more than 93% similar to the species test sequence (the median similarity of the SSU ribotags in the soil sample to reference sequences in the LSUrdb). Note that no order level in the Crenarchaeote 54d9 is defined. (0.74 MB TIF) [file pone.0002527.s005.tif]

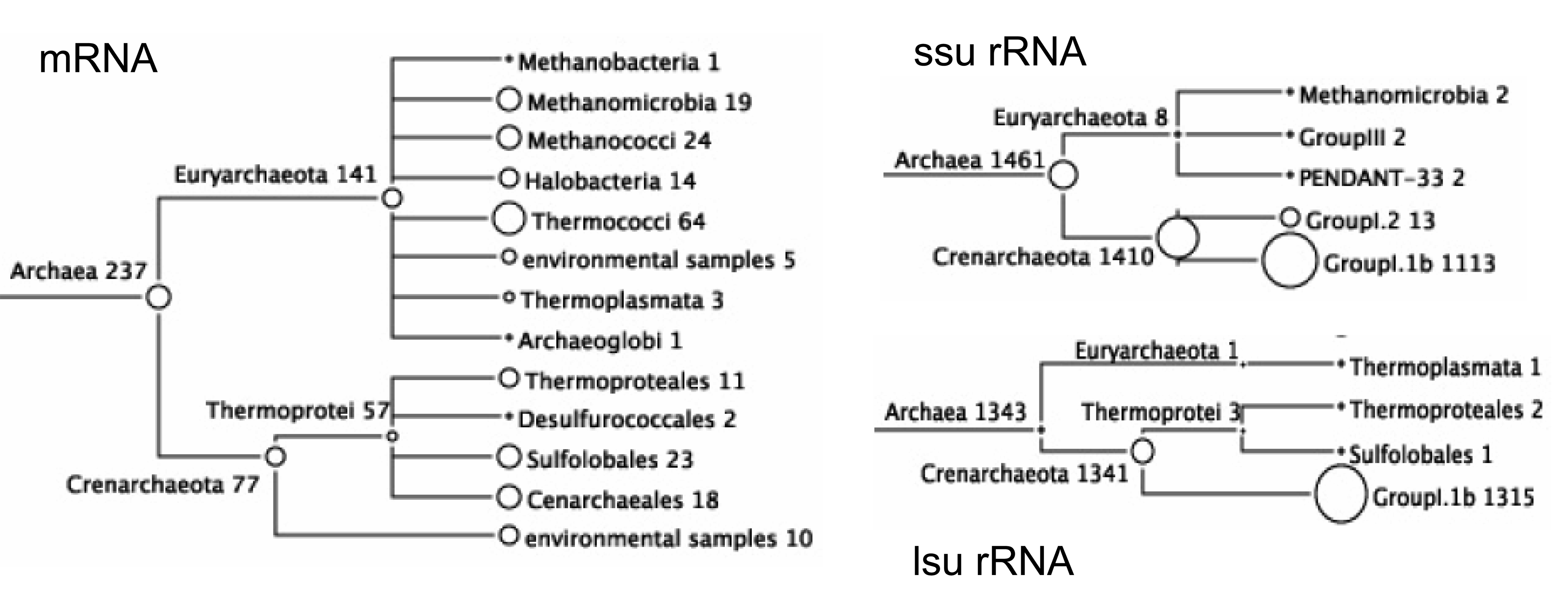

Supplement: Figure S5 — MEGAN comparison of archaeal rRNA based and mRNA based community profile. The taxonomic affiliation of an RNA-tag is based on the Blast hits within 10% of the top Blast Bit score. (0.94 MB TIF) [file pone.0002527.s006.tif]

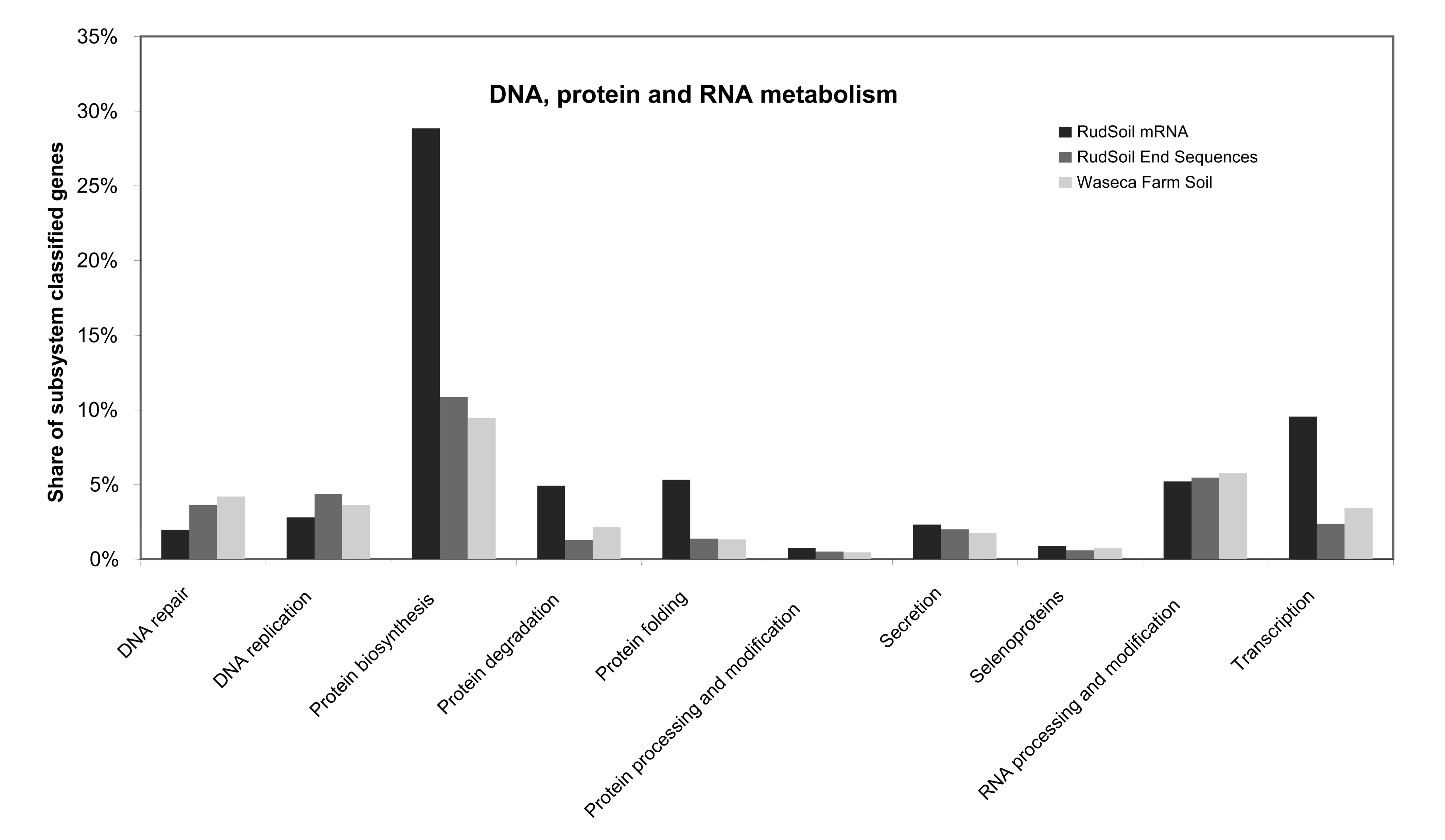

Supplement: Figure S6 — Functional analysis of DNA, protein and RNA metabolism subsystems in Rudsoil mRNA-tags, fosmid-derived end sequences from DNA of the same community (Treusch et al., 2004) and shotgun-cloned DNA from a farm soil community (Tringe et al., 2004). All three datasets were subjected to automated analysis using the MG-RAST annotation procedure at the SEED (http://metagenomics.theseed.org). (0.26 MB TIF) [file pone.0002527.s007.tif]

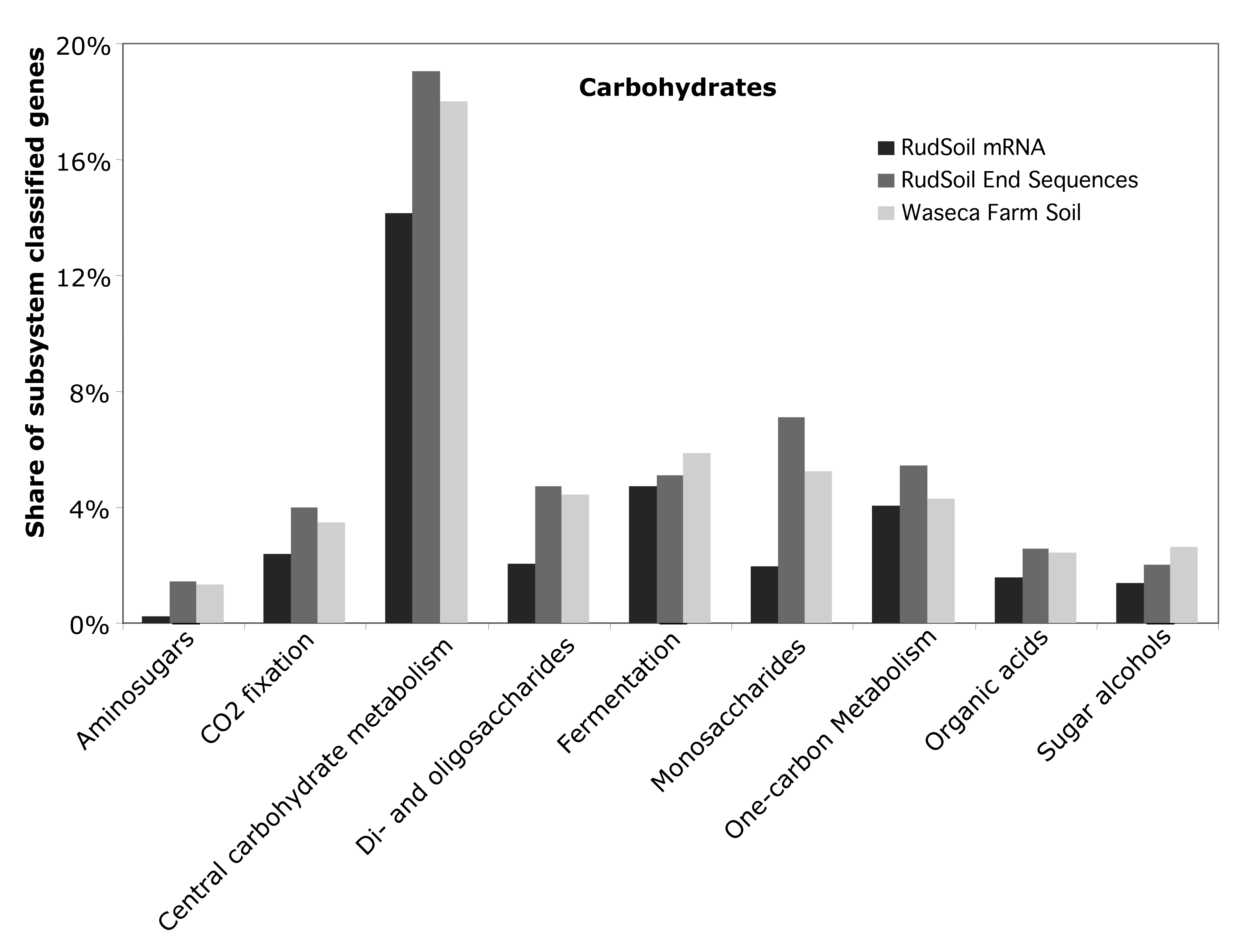

Supplement: Figure S7 — Functional analysis of carbohydrate metabolism subsystems in Rudsoil mRNA-tags, fosmid-derived end sequences from DNA of the same community (Treusch, 2004) and shotgun-cloned DNA from a farm soil community (Tringe, 2004). All three datasets were subjected to automated analysis using the MG-RAST annotation procedure at the SEED (http://metagenomics.theseed.org). (0.20 MB TIF) [file pone.0002527.s008.tif]

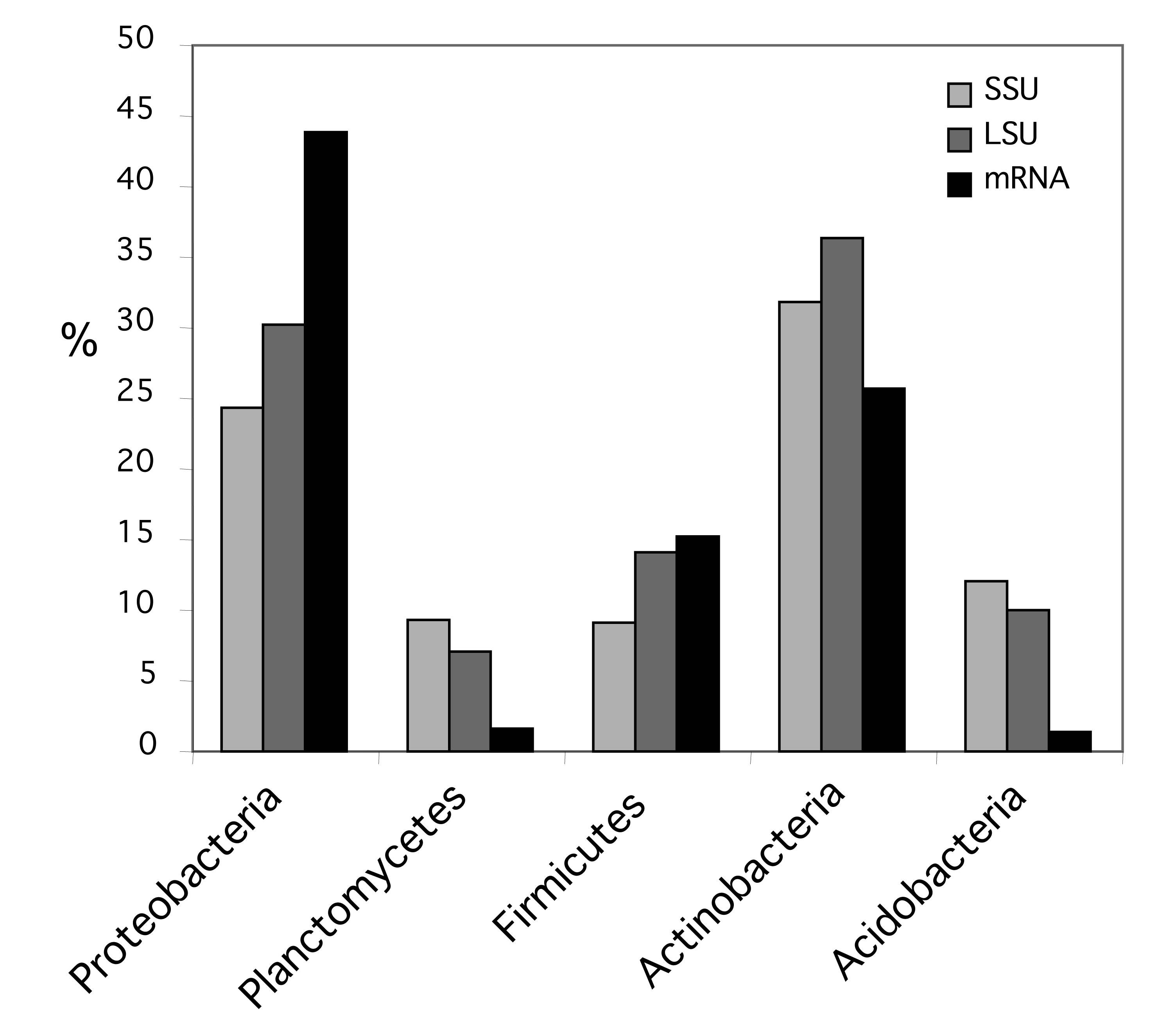

Supplement: Figure S8 — Fraction of phylum-resolution SSU and LSU ribo-tags (in %) affiliated to the five numerically dominant bacterial phyla (>5% of bacterial ribo-tags) compared to mRNA-tag derived fraction for each phylum. (0.15 MB TIF) [file pone.0002527.s009.tif]

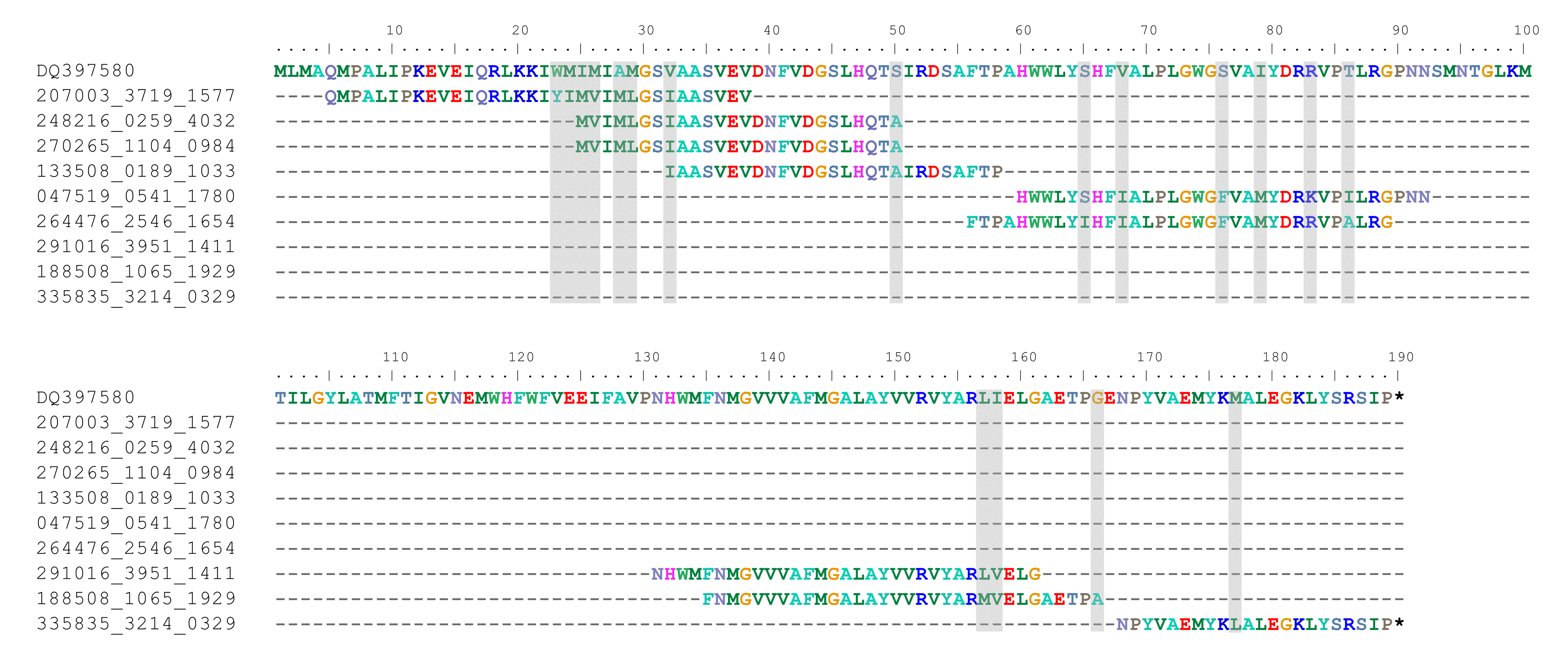

Supplement: Figure S9 — The soil crenarchaeota “composite” AmoC protein. Alignment of 9 amoC mRNA-tags (translated into amino acid sequence) against the AmoC protein of Cenarchaeum symbiosum (DQ397580), a member of the marine groupI.1a Crenarchaeota. Mismatches between the sequences are shaded gray. The nine mRNA-tags form three fragments which cover 77% of the C. symbiosum AmoC with 88% sequence identity. (1.78 MB TIF) [file pone.0002527.s010.tif]
