# Supplementary material for: Simultaneous Assessment of Soil Microbial Community Structure and Function through Analysis of the Meta-Transcriptome
Source: PLoS One. 2008 Jun 25;3(6):e2527. doi: 10.1371/journal.pone.0002527 (PMC2424134; doi:10.1371/journal.pone.0002527)
Supplement: Table S1 — Schematic overview of the sequence content of SSUrdb and LSUrdb. (0.03 MB DOC) [file pone.0002527.s011.doc]

**Supplementary table ST1:** Schematic overview of the sequence content of SSUrdb and SUrdb.

|  | SSUrdb | LSUrdb |
| --- | --- | --- |
| total | 137,160 | 6,247 |
| Bacteria | 111,473 | 2,759 |
| Archaea | 1,490 | 130 |
| Eukaryota (total)  Plastids  Mitochondria | 24,197  641  331 | 3,358  491  122 |

The total number of sequences and the number affiliated to Bacteria, Archaea and Eukaryota (including plastid and mitochondrial sequences) is given. Note that the SSUrdb contains approximately 22 times more sequences than the LSUrdb. In addition, the relative proportion of the three domains of life differs in both databases.
