# Supplementary material for: Simultaneous Assessment of Soil Microbial Community Structure and Function through Analysis of the Meta-Transcriptome
Source: PLoS One. 2008 Jun 25;3(6):e2527. doi: 10.1371/journal.pone.0002527 (PMC2424134; doi:10.1371/journal.pone.0002527)
Supplement: Table S2 — Archaeal candidate divisions implemented into the NCBI taxonomy. (0.05 MB DOC) [file pone.0002527.s012.doc]

**Supplementary Table ST2:** Archaeal candidate divisions implemented into the NCBI taxonomy.

| Phylum | Candidate division | SSUrdb | LSUrdb |
| --- | --- | --- | --- |
| Crenarchaeota | THSC1 | 13 | - |
|  | YNPFFA | 8 | - |
|  | MarBenthGpC | 42 | - |
|  | MarBenthGpB-DHVC1 | 26 | - |
|  | GroupI.2 | 8 | - |
|  | GroupI.1c | 22 | - |
|  | GroupI.1b | 56 | 2 |
|  | SAGMCG-1 | 10 | - |
|  | GroupI.1a | 103 | 6 |
| Euryarchaeota | DHVE4 | 8 | - |
|  | SAGMA1 | 10 | - |
|  | DHVE1 | 7 | - |
|  | Thermoplasmata:  uncultured Thermoplasmata | 10 | - |
|  | GroupII | 21 | 1 |
|  | SAGMA-ST | 2 | - |
|  | GroupIII | 15 | - |
|  | VADIN | 20 | - |
|  | MarBenthGpE | 13 | - |
|  | DHVE6 | 12 | - |
|  | PENDANT-33 | 17 | - |
|  | SA1 | 10 | - |
|  | DHVE3 | 12 | - |
|  | Rice cluster1 | 5 | 6 |
|  | ANME1 | 44 | 7 |
|  | ANME2 | 40 | 1 |
|  | ARC1 | 9 | - |
| Korarchaeota |  | 8 | - |
| Ancient archaeal group (AAG) |  | 4 | - |

The numbers in SSUrdb and LSUrdb refer to the number of sequences in the reference databases for each taxon.
