# Supplementary material for: Simultaneous Assessment of Soil Microbial Community Structure and Function through Analysis of the Meta-Transcriptome
Source: PLoS One. 2008 Jun 25;3(6):e2527. doi: 10.1371/journal.pone.0002527 (PMC2424134; doi:10.1371/journal.pone.0002527)
Supplement: Table S3 — Result of BLASTN of SSU rRNA test dataset derived from 43 species against SSUrdb. (0.12 MB DOC) [file pone.0002527.s013.doc]

**Supplementary Table ST3:** Result of BLASTN of SSU rRNA test dataset derived from 43 species against SSUrdb (see right column for species name).

| SSUrdb | Domain | Phylum | Class | Order | Family | Genus | Species |
| --- | --- | --- | --- | --- | --- | --- | --- |
| Cellular | Bacteria | Proteobacteria | Alpha 390/400 = 96.8% | Rhizobiales 287/400 = 71.8% | Bradyrhizobiaceae | Bradyrhizobium | B. japonicum |
| organisms | 6390/6400 | 2670/2800 |  |  |  | Nitrobacter | N. winogradskyi |
| 10/8600 | 99.8% | 94.5% | Beta 550/600 = 91.6% | Burkholderiales 187/200 = 93.5% | Burkholderiaceae | Burkholderia | B. cepaia |
|  |  |  |  | Nitrosomonadales 313/400 = 78.3% | Nitrosomonadaceae | Nitrosomonas | N. eutropha |
|  |  |  |  |  |  | Nitrosospira | N. multiformis |
|  |  |  | Gamma 1212/1400 = 86.6% | Enterobacteriales 584/800 = 73%% | Enterobacteriaceae | Salmonella | S. typhimurium |
|  |  |  |  |  |  | Shigella | S. dysenteriae |
|  |  |  |  |  |  | Yersinia | Y. pestis |
|  |  |  |  |  |  | Escherichia | E. coli K12 |
| Color coding: |  |  |  | Pseudomonadales 299/400 = 75% | Pseudomonadaceae | Pseudomonas | P. fluorescens |
| 100% |  |  |  |  | Moraxacellaceae | Acinetobacter | A. baumannii |
| <100%-95% |  |  |  | Chromatiales 161/200 = 80.5% | Chromatiaceae | Nitrosococcus | N. oceani |
| <95%-90% |  |  | Delta 200/200 = 100% | Myxococcales 191/200 = 95.5% | Myxococcaceae | Myxococcus | M. xanthus |
| <90%-80% |  |  | Epsilon 187/200 = 93.5% | Campylobacterales 187/200=93.5% | Helicobacteraceae | Wolinella | W.succinogenes |
| <80%-70% |  | Spirochaetes 197/200 = 98.5% | Spirochaetes 197/200 = 98.5% | Spirochaetales 197/200 = 98.5% | Spirochaetaceae | Borrelia | B. burgdorferi |
| <70% |  | Cyanobacteria 194/200 = 97% |  | Nostocales 75/200 = 37.5% | Nostocaceae | Anabaena | A. variabilis |
|  |  | Firmicutes 595/600 = 99.2% | Bacilli | Bacillales 192/200 = 96% | Bacillaceae | Bacillus | B. anthracis |
|  |  |  | Clostridia | Clostridiales 194/200 = 97% | Clostridiaceae | Clostridium | C. tetani |
|  |  |  | Mollicutes | Mycoplasmatales 200/200 = 100% | Mycoplasmataceae | Mycoplasma | M. genitalum |
|  |  | Aquificae 191/200 = 95.5% | Aquificae | Aquificales 191/200 = 95.5% | Aquificaceae | Aquifex | A. aeolicus |
|  |  | Chlamydiae 198/200 = 99% | Chlamydiae | Chlamydiales 178/200 = 89% | Parachlamydiaceae | Cand. Protochlamydia | P. amoebophila |
|  |  | Bacteroidetes 396/400 = 99% | Bacteroidetes | Bacteroidales 196/200 = 98% | Bacteroidaceae | Bacteroides | B. thetaiomicron |
|  |  |  | Flavobacteria | Flavobacteriales 42/200 = 21% | Flavobacteriaceae | Flavobacterium | F. johnsoniae |
|  |  | Chlorobi 197/200 = 98.5 | Chlorobia | Chlorobiales 66/200 = 33% | Chlorobiaceae | Chlorobaculum | Chl. tepidum |
|  |  | Chloroflexi 181/200 = 91.5% | Dehalococcidetes 166/200=83% |  |  | Dehalococcoides | D. sp. CBDB1 |
|  |  | Deinococcus-Thermus 96% | Deinococci | Deinococcales 191/200 = 96% | Deinococcaceae | Deinococcus | D. geothermalis |
|  |  | Planctomycetes | Planctomycetacia | Planctomycetales | Planctomycetaceae | Rhodopirellula | R. baltica |
|  |  | 391/400 = 97.8% |  | 391/400 = 97.8% | Unclass. Planct. | Candidatus Kuenenia | K. stuttgartiensis |
|  |  | Actinobacteria 400/400= 100% | Actinobacteria | Actinomycetales 377/400 = 94.3% | Mycobacteriaceae | Mycobacterium | M. leprae |
|  |  |  |  |  | Streptomycetaceae | Streptomyces | S. coelicolor |
|  |  | Acidobacteria 200/200 =100% | Solibacteres | Solibacterales 24/200 = 12% | Solibacteraceae | Solibacter | S. usitatus |
|  |  | Thermotogae 199/200= 99.5% | Thermotogae | Thermotogales 199/200 = 99.5% | Thermotogaceae | Thermotoga | T. maritima |
|  | Archaea | Euryarchaeota 400/400=100% | Methanomicrobia 171/200=85.5 | Methanosarcinales170/200 | Methanosarcinaceae | Methanosarcina | M. mazei |
|  | 1000/1000 |  | Halobacteria 200/200 = 100% | Halobacteriales 200/200 = 100% | Halobacteriaceae | Haloquadratum | H. walsbyi |
|  | 100% | Crenarchaeota 600/600=100% | Thermoprotei 200/200 = 100% | Sulfolobales 199/200 = 99.5% | Sulfolobaceae | Sulfolobus | S. solfataricus |
|  |  |  | GroupI.1a 192/200 = 96% |  |  | Cenarchaeum | C. symbiosum |
|  |  |  | GroupI.1b 176/200 = 88% |  |  |  | Unc. Cren. 54d9 |
|  | Eukaryota | Viridiplantae 372/400 = 93% | Spermatophyta 325/400=81.3% | Poales 30/200 = 15% | Poaceae | Oryza | O. sativa |
|  | 1198/1200 |  |  | Brassicales 43/200 = 21.5% | Brassicaceae | Arabidopsis | A. thaliana |
|  | 99.8% | Fungi 193/200 = 96.5% | Ascomycota 173/200 = 86.5% | Saccharomycetales 123/200=61.5 | Saccharomycetaceae | Saccharomyces | S. cerevisiae |
|  |  | Alveolata 196/200 = 98% | Apicomplexa 196/200 = 98% | Haemosporida 196/200 = 98% |  | Plasmodium | P. falciparum |
|  |  | Metazoa 400/400 = 100% | Arthropoda 195/200 = 97.5% | Diptera 192/200 = 96% | Drosophilidae | Drosophila | D. melanogaster |
|  |  |  | Nematoda 200/200 = 100% | Rhabditida 120/200 = 60% | Rhabditidea | Cenorhabditis | C. elegans |

The data set consisted of 8,600 simulated ribo-tags of 100bp length (200 randomly generated ones from each species). The ribo-tags were taxonomically affiliated according to a BLASTN bit score of 86, and BLASTN hits within the top ten percent of Bit score were included in the taxonomic analysis with MEGAN. The number and percentage of ribo-tags for each species are given at different taxonomic resolution, from the domain until the order level. The color coding refers to different percentages of ribo-tags correctly affiliated at a given taxonomic level. No ribo-tag was falsely assigned. 10 ribo-tags were affiliated as "cellular organisms", i.e. could not be assigned to respective domain of live.
