# Supplementary material for: Simultaneous Assessment of Soil Microbial Community Structure and Function through Analysis of the Meta-Transcriptome
Source: PLoS One. 2008 Jun 25;3(6):e2527. doi: 10.1371/journal.pone.0002527 (PMC2424134; doi:10.1371/journal.pone.0002527)
Supplement: Table S4 — Result of BLASTN of LSU rRNA test dataset derived from 43 species against LSUrdb. (0.12 MB DOC) [file pone.0002527.s014.doc]

**Supplementary Table ST4:** Result of BLASTN of LSU rRNA test dataset derived from 43 species against LSUrdb (see very column for species name).

| LSUrdp | Domain | Phylum | Class | Order | Family | Genus | Species |
| --- | --- | --- | --- | --- | --- | --- | --- |
| Cellular | Bacteria | Proteobacteria | Alpha 388/400 = 97% | Rhizobiales 359/400 =89.8% | Bradyrhizobiaceae | Bradyrhizobium | B. japonicum |
| organisms | 6382/6400 | 2779/2800 |  |  |  | Nitrobacter | N. winogradskyi |
| 18/8600 | 99.7% | 99.3% | Beta 563/600 = 93.8% | Burkholderiales 170/200 = 85% | Burkholderiaceae | Burkholderia | B. cepaia |
|  |  |  |  | Nitrosomonadales 284/400 = 71% | Nitrosomonadaceae | Nitrosomonas | N. eutropha |
|  |  |  |  |  |  | Nitrosospira | N. multiformis |
|  |  |  | Gamma 1381/1400 = 98.6% | Enterobacteriales 681/800 = 85.1% | Enterobacteriaceae | Salmonella | S. typhimurium |
|  |  |  |  |  |  | Shigella | S. dysenteriae |
|  |  |  |  |  |  | Yersinia | Y. pestis |
|  |  |  |  |  |  | Escherichia | E. coli K12 |
| Color coding: |  |  |  | Pseudomonadales 307/400 =76.8% | Pseudomonadaceae | Pseudomonas | P. fluorescens |
| 100% |  |  |  |  | Moraxacellaceae | Acinetobacter | A. baumannii |
| <100%-95% |  |  |  | Chromatiales 187/200 = 93.5% | Chromatiaceae | Nitrosococcus | N. oceani |
| <95%-90% |  |  | Delta 191/200 = 95.5% | Myxococcales 191/200 = 95.5% | Myxococcaceae | Myxococcus | M. xanthus |
| <90%-80% |  |  | Epsilon 200/200 = 100% | Campylobacterales 200/200 =100% | Helicobacteraceae | Wolinella | W.succinogenes |
| <80%-70% |  | Spirochaetes 197/200 = 98.5% | Spirochaetes 197/200 = 98.5% | Spirochaetales 197/200 = 98.5% | Spirochaetaceae | Borrelia | B. burgdorferi |
| <70% |  | Cyanobacteria 184/200 = 92% |  | Nostocales 171/200 = 85.5% | Nostocaceae | Anabaena | A. variabilis |
|  |  | Firmicutes 597/600 = 99.5% | Bacilli | Bacillales 192/200 = 96% | Bacillaceae | Bacillus | B. anthracis |
|  |  |  | Clostridia | Clostridiales 183/200 = 91.5% | Clostridiaceae | Clostridium | C. tetani |
|  |  |  | Mollicutes | Mycoplasmatales 200/200 = 100% | Mycoplasmataceae | Mycoplasma | M. genitalum |
|  |  | Aquificae 200/200 = 100% | Aquificae | Aquificales 200/200 = 100% | Aquificaceae | Aquifex | A. aeolicus |
|  |  | Chlamydiae 199/200 = 99.5% | Chlamydiae | Chlamydiales 199/200 = 99.5% | Parachlamydiaceae | Cand. Protochlamydia | P. amoebophila |
|  |  | Bacteroidetes 394/400 =98.5% | Bacteroidetes | Bacteroidales 188/200 = 94% | Bacteroidaceae | Bacteroides | B. thetaiomicron |
|  |  |  | Flavobacteria | Flavobacteriales 189/200 = 94.5% | Flavobacteriaceae | Flavobacterium | F. johnsoniae |
|  |  | Chlorobi 197/200 = 98.5 | Chlorobia | Chlorobiales 197/200 = 98.5% | Chlorobiaceae | Chlorobaculum | Chl. tepidum |
|  |  | Chloroflexi 200/200 = 100% | Dehalococcidetes |  |  | Dehalococcoides | D. sp. CBDB1 |
|  |  | Deinococcus-Thermus =98.5% | Deinococci | Deinococcales 197/200 = 98.5% | Deinococcaceae | Deinococcus | D. geothermalis |
|  |  | Planctomycetes 400/400=100 | Planctomycetacia | Planctomycetales 400/400 = 100% | Planctomycetaceae | Rhodopirellula | R. baltica |
|  |  |  |  |  | Unclass Planctom. | Candidatus Kuenenia | K. stuttgartiensis |
|  |  | Actinobacteria 400/400 =100% | Actinobacteria | Actinomycetales 398/400 = 99.5% | Mycobacteriaceae | Mycobacterium | M. leprae |
|  |  |  |  |  | Streptomycetaceae | Streptomyces | S. coelicolor |
|  |  | Acidobacteria 194/200 =97% | Solibacteres | Solibacterales 186/200 = 93% | Solibacteraceae | Solibacter | S. usitatus |
|  |  | Thermotogae 197/200=98.5% | Thermotogae | Thermotogales 197/200=98.5% | Thermotogaceae | Thermotoga | T. maritima |
|  | Archaea | Euryarchaeota 400/400=100% | Methanomicrobia 194/200= 97% | Methanosarcinales 194/200 = 97% | Methanosarcinaceae | Methanosarcina | M. mazei |
|  | 1000/1000 |  | Halobacteria 200/200 = 100% | Halobacteriales 200/200 = 100% | Halobacteriaceae | Haloquadratum | H. walsbyi |
|  | 100% | Crenarchaeota 600/600=100% | Thermoprotei 200/200 = 100% | Sulfolobales 200/200 = 100% | Sulfolobaceae | Sulfolobus | S. solfataricus |
|  |  |  | GroupI.1a 197/200 = 98.5% |  |  | Cenarchaeum | C. symbiosum |
|  |  |  | GroupI.1b 199/200 = 99.5% |  |  |  | Unc. Cren. 54d9 |
|  | Eukaryota | Viridiplantae 375/400 = 93.8% | Spermatophyta 350/400 =87.5% | Poales 75/200 = 37.5% | Poaceae | Oryza | O. sativa |
|  | 1190/1200 |  |  | Brassicales 68/200 = 34% | Brassicaceae | Arabidopsis | A. thaliana |
|  | 99.2% | Fungi 187/200 = 93.5% | Ascomycota 158/200 = 79% | Saccharomycetales 144/200 = 72% | Saccharomycetaceae | Saccharomyces | S. cerevisiae |
|  |  | Alveolata 186/200 = 93% | Apicomplexa 186/200 = 93% | Haemosporida 185/200 = 92.5% |  | Plasmodium | P. falciparum |
|  |  | Metazoa 386/400 = 96.5% | Arthropoda 188/200 = 94% | Diptera 188/200 = 94% | Drosophilidae | Drosophila | D. melanogaster |
|  |  |  | Nematoda 195/200 = 97.5% | Rhabditida 194/200 = 97% | Rhabditidea | Cenorhabditis | C. elegans |

The data set consisted of 8,600 simulated ribo-tags (200 randomly generated ones from each species). The ribo-tags were taxonomically affiliated according to a BLASTN-bit score of 86, and BLASTN hits within the top ten percent of Bit score were included in the taxonomic analysis with MEGAN. The number and percentage of ribo-tags for each species are given at different taxonomic resolution, from the domain until the order level. The color coding refers to different percentages of ribo-tags correctly affiliated at a given taxonomic level. No ribo-tag was falsely-assigned. 18 ribo-tags were affiliated as "cellular organisms", i.e. could not be assigned to the correct domain of live.
