# Supplementary material for: Simultaneous Assessment of Soil Microbial Community Structure and Function through Analysis of the Meta-Transcriptome
Source: PLoS One. 2008 Jun 25;3(6):e2527. doi: 10.1371/journal.pone.0002527 (PMC2424134; doi:10.1371/journal.pone.0002527)
Supplement: Table S5 — Sensitivity analysis results for the SSUrdb and LSUrdb. (0.14 MB DOC) [file pone.0002527.s015.doc]

**Supplementary Table ST5:** Sensitivity analysis results for the SSurdb and LSUrdb.

| ***A. Simulated ribotags correctly assigned per taxonomic level in SSUrdb (unfiltered)*** | | | | | | | |
| --- | --- | --- | --- | --- | --- | --- | --- |
|  | Order | Class | Phylum | Domain | Cell. Orgranism | Unassigned | Incorrectly assigned |
| Escherichia coli K12 | 154 | 198 | 200 | 200 | 200 | 0 | 0 |
| Myxococcus xanthus | 195 | 200 | 200 | 200 | 200 | 0 | 0 |
| Borrelia burgdorferi | 197 | 197 | 200 | 200 | 200 | 0 | 0 |
| Aquifex aeolicus | 185 | 185 | 185 | 200 | 200 | 0 | 0 |
| Rhodopirellula baltica | 192 | 192 | 192 | 200 | 200 | 0 | 0 |
| Uncultured crenarchaeote 54d9 | - | 181 | 200 | 200 | 200 | 0 | 0 |
| Saccharomyces cerevisiae | 127 | 157 | 166 | 200 | 200 | 0 | 0 |
| *Total* | *1050* | *1310* | *1343* | *1400* | *1400* |  |  |
| *Share* | *88%* | *94%* | *96%* | *100%* | *100%* |  |  |
|  |  |  |  |  |  |  |  |
| ***B. Simulated ribotags correctly assigned per taxonomic level in SSUrdb filtered from 98% similar*** | | | | | | | |
|  | Order | Class | Phylum | Domain | Cell. Organism | Unassigned | Incorrectly assigned |
| Escherichia coli K12 | 112 | 194 | 196 | 200 | 200 | 0 | 0 |
| Myxococcus xanthus | 191 | 199 | 200 | 200 | 200 | 0 | 0 |
| Borrelia burgdorferi | 197 | 197 | 197 | 200 | 200 | 0 | 0 |
| Aquifex aeolicus | 184 | 184 | 184 | 200 | 200 | 0 | 0 |
| Rhodopirellula baltica | 192 | 192 | 192 | 200 | 200 | 0 | 0 |
| Uncultured crenarchaeote 54d9 | - | 178 | 200 | 200 | 200 | 0 | 0 |
| Saccharomyces cerevisiae | 63 | 142 | 161 | 200 | 200 | 0 | 0 |
| *Total* | *939* | *1286* | *1330* | *1400* | *1400* |  |  |
| *Share* | *78%* | *92%* | *95%* | *100%* | *100%* |  |  |
|  |  |  |  |  |  |  |  |
| ***C. Simulated ribotags correctly assigned per taxonomic level in SSUrdb filtered from 86% similar*** | | | | | | | |
|  | Order | Class | Phylum | Domain | Cell. Organism | Unassigned | Incorrectly assigned |
| Escherichia coli K12 | 70 | 124 | 196 | 200 | 200 | 0 | 0 |
| Myxococcus xanthus | 8 | 28 | 34 | 160 | 160 | 24 | 15 |
| Borrelia burgdorferi | 58 | 58 | 58 | 144 | 153 | 40 | 7 |
| Aquifex aeolicus | 83 | 83 | 128 | 128 | 145 | 42 | 12 |
| Rhodopirellula baltica | 56 | 56 | 56 | 75 | 75 | 122 | 3 |
| Uncultured crenarchaeote 54d9 | - | 89 | 150 | 162 | 162 | 11 | 27 |
| Saccharomyces cerevisiae | 75 | 75 | 103 | 193 | 193 | 6 | 1 |
| *Total* | *350* | *513* | *725* | *1062* | *1088* | *245* | *65* |
| *Share* | *29%* | *37%* | *52%* | *76%* | *78%* | *18%* | *5%* |
|  |  |  |  |  |  |  |  |
|  |  |  |  |  |  |  |  |
| ***D. Simulated ribotags correctly assigned per taxonomic level in LSUrdb (unfiltered)*** | | | | | | | |
|  | Order | Class | Phylum | Domain | Cell. Organism | Unassigned | Incorrectly assigned |
| Escherichia coli K12 | 171 | 200 | 200 | 200 | 200 | 0 | 0 |
| Myxococcus xanthus | 190 | 191 | 192 | 200 | 200 | 0 | 0 |
| Borrelia burgdorferi | 198 | 198 | 198 | 200 | 200 | 0 | 0 |
| Aquifex aeolicus | 200 | 200 | 200 | 200 | 200 | 0 | 0 |
| Rhodopirellula baltica | 200 | 200 | 200 | 200 | 200 | 0 | 0 |
| Uncultured crenarchaeote 54d9 | - | 199 | 200 | 200 | 200 | 0 | 0 |
| Saccharomyces cerevisiae | 149 | 149 | 156 | 200 | 200 | 0 | 0 |
| *Total* | *1108* | *1337* | *1346* | *1400* | *1400* |  |  |
| *Share* | *92%* | *96%* | *96%* | *100%* | *100%* |  |  |
|  |  |  |  |  |  |  |  |
| ***E. Simulated ribotags correcty assigned per taxonomic level in LSUrd filtered from 93% similar*** | | | | | | | |
|  | Order | Class | Phylum | Domain | Cell. Organism | Unassigned | Incorrectly assigned |
| Escherichia coli K12 | 134 | 198 | 200 | 200 | 200 | 0 | 0 |
| Myxococcus xanthus | 167 | 171 | 174 | 186 | 186 | 14 | 0 |
| Borrelia burgdorferi | 1 | 29 | 29 | 62 | 76 | 84 | 40 |
| Aquifex aeolicus | 0 | 0 | 0 | 21 | 26 | 152 | 22 |
| Rhodopirellula baltica | 157 | 157 | 157 | 157 | 158 | 41 | 1 |
| Uncultured crenarchaeote 54d9 | - | 157 | 159 | 159 | 159 | 41 | 0 |
| Saccharomyces cerevisiae | 126 | 126 | 134 | 191 | 191 | 9 | 0 |
| *Total* | *585* | *838* | *853* | *976* | *996* | *341* | *63* |
| *Share* | *49%* | *60%* | *61%* | *70%* | *71%* | *24%* | *5%* |
|  |  |  |  |  |  |  |  |

SSU and LSU test sequences from seven species were used to generate random 100bp simulated ribo-tags (200 from each species for SSU and LSU). These were then compared to the complete SSUrdb (a) and LSUrdb (d) using BLASTN and assigned using MEGAN. In addition to using the full databse, filtering was carried out, and the simulated ribo-tags were aligned to the filtered database before assignment in MEGAN. Filtering was carried out by aligning the contained database sequences to the full length test sequence and removing them if the similarity was higher than the treshold. The SSUrdb was filtered at 98% (b) and 86% (c), while the LSUrdb was filtered at 93% (e). These percentages correspond to the median (b, e) and lowest decile (c) similarity between assigned ribotags in the sample and their best scoring BLASTN hits. For each database, the cumulative number of assignments per taxonomic level is displayed, incl. the root level “cellular organisms” meaning that a ribo-tag was assigned as SSU or LSU but could not be assigned to a specific domain of life. In addition, the number of unassigned ribo-tags (false negatives) and incorrectly assigned tags (false positives) are displayed.
