# Supplementary material for: Simultaneous Assessment of Soil Microbial Community Structure and Function through Analysis of the Meta-Transcriptome
Source: PLoS One. 2008 Jun 25;3(6):e2527. doi: 10.1371/journal.pone.0002527 (PMC2424134; doi:10.1371/journal.pone.0002527)
Supplement: Table S6 — The bacterial community structure in soil. (0.11 MB DOC) [file pone.0002527.s016.doc]

**Supplementary Table ST6:** The bacterial community structure in soil.

| Ribo-tags of bacterial phyla | SSUrdb | LSUrdb | SSU% | LSU% | mean% |
| --- | --- | --- | --- | --- | --- |
| Ribo-tags with phylum-resolution | 70268 | 70848 | 100 | 100 | 100 |
| Proteobacteria | 17088 | 21390 | 24.3 | 30.2 | 27.3 |
| Planctomycetes | 6617 | 5417 | 9.4 | 7.7 | 8.5 |
| Firmicutes | 6437 | 9789 | 9.2 | 13.8 | 11.5 |
| Actinobacteria | 22241 | 25274 | 31.7 | 35.7 | 33.7 |
| Acidobacteria | 8359 | 7077 | 11.9 | 10.0 | 10.9 |
| Chloroflexi | 3343 | 207 | 4.76 | 0.29 | 2.53 |
| Bacteroidetes | 1442 | 653 | 2.05 | 0.92 | 1.49 |
| Chlorobi | 11 | 21 | 0.016 | 0.030 | 0.023 |
| Spirochaetes | 21 | 22 | 0.030 | 0.031 | 0.030 |
| Deinococcus-Thermus | 9 | 62 | 0.013 | 0.088 | 0.050 |
| Fusobacteria | 0 | 21 | 0.000 | 0.030 | 0.015 |
| Cyanobacteria | 44 | 118 | 0.063 | 0.167 | 0.115 |
| Deferribacteres | 1 | -* | 0.001 | - | - |
| Thermotogae | 1 | 53 | 0.001 | 0.075 | 0.038 |
| Fibrobacteres | 22 | 3 | 0.031 | 0.004 | 0.018 |
| Dictyoglomi | 3 | 11 | 0.004 | 0.016 | 0.010 |
| Nitrospirae | 277 | - | 0.394 | - | - |
| Chlamydiae | 62 | 156 | 0.088 | 0.220 | 0.154 |
| Verrucomicrobia | 1256 | 361 | 1.787 | 0.510 | 1.149 |
| Lentisphaerae | 4 | - | 0.006 | - | - |
| Gemmatimonadetes | 1124 | - | 1.600 | - | - |
| Candidate phyla (see below) | 1803 | - | 2.566 | - | - |
| OP3 | 7 | - | 0.010 | - | - |
| OP8 | 213 | - | 0.303 | - | - |
| OP10 | 218 | - | 0.310 | - | - |
| OP11 | 18 | - | 0.026 | - | - |
| WS3 | 44 | - | 0.063 | - | - |
| WS2 | 1 | - | 0.001 | - | - |
| WS6 | 2 | - | 0.003 | - | - |
| OD1 | 2 | - | 0.003 | - | - |
| TG1 | 27 | - | 0.038 | - | - |
| TM6 | 13 | - | 0.019 | - | - |
| TM7 | 27 | - | 0.038 | - | - |
| OS-K | 2 | - | 0.003 | - | - |
| BRC1 | 5 | - | 0.007 | - | - |
| KSB1 | 1 | - | 0.001 | - | - |
| KSB2 | 3 | - | 0.004 | - | - |
| NKB19 | 224 | - | 0.319 | - | - |
| WYO | 25 | - | 0.036 | - | - |
| SPAM | 680 | - | 0.968 | - | - |
| TG3 | 3 | - | 0.004 | - | - |
| VC2 | 263 | - | 0.374 | - | - |

*- no value available, because no sequence of the respective taxon present in reference database.

The absolute and relative distribution of ribo-tags is given. All bacterial ribo-tags with a taxonomic resolution until the phylum-level or higher were included. The ribo-tags were taxonomically affiliated according to a BLASTN-bit score of 86, and BLASTN hits within the top ten percent of Bit score were taxonomically analysed.
