# Supplementary material for: Simultaneous Assessment of Soil Microbial Community Structure and Function through Analysis of the Meta-Transcriptome
Source: PLoS One. 2008 Jun 25;3(6):e2527. doi: 10.1371/journal.pone.0002527 (PMC2424134; doi:10.1371/journal.pone.0002527)
Supplement: Table S7 — The archaeal community structure in soil. (0.04 MB DOC) [file pone.0002527.s017.doc]

**Supplementary Table ST7:** The archaeal community structure in soil.

| Ribo-tags of archaeal phyla | SSUrdb | LSUrdb | SSUrdb% | LSUrdb% | mean% |
| --- | --- | --- | --- | --- | --- |
| Ribo-tags with phylum-resolution | 1132 | 1319 | 100 | 100 | 100 |
| GroupI.1b (Crenarchaeota) | 1113 | 1315 | 98.3 | 99.6 | 99.0 |
| GroupI.2 (Crenarchaeota) | 13 | -* | 1.15 | - | - |
| Thermoprotei (Crenarchaeota) | 0 | 3 | 0 | 0.23 | 0.115 |
| Methanomicrobia (Euryarchaeota) | 2 | 0 | 0.18 | 0 | 0.09 |
| GroupIII (Euryarchaeota) | 2 | - | 0.18 | - | - |
| PENDANT-33 (Euryarchaeota) | 2 | - | 0.18 | - | - |
| Thermoplasma (Euryarchaeota) | 0 | 1 | 0 | 0.08 | 0.04 |

*- no value available, because no sequence of the respective taxon present in reference database.

The absolute and relative distribution of archaeal ribo-tags is given. All archaeal ribo-tags with a taxonomic resolution to the phylum-level or higher were included. The ribo-tags were taxonomically affiliated according to a BLASTN-bit score of 86, and BLASTN hits within the top ten percent of Bit score were taxonomically analysed.
