# Supplementary material for: Simultaneous Assessment of Soil Microbial Community Structure and Function through Analysis of the Meta-Transcriptome
Source: PLoS One. 2008 Jun 25;3(6):e2527. doi: 10.1371/journal.pone.0002527 (PMC2424134; doi:10.1371/journal.pone.0002527)
Supplement: Table S8 — Statistics from functional analysis of putative mRNA-tags. (0.03 MB DOC) [file pone.0002527.s018.doc]

**Supplementary Table ST8:** Statistics from functional analysis of putative mRNA-tags.

| Dataset | RudSoil  mRNA-tags | RudSoil Genomic^*^ | Waseca Farm Soil Genomic^**^ |
| --- | --- | --- | --- |
| Seq. Size (kbp) | 2,077 | 4,294 | 144,838 |
| Fragments | 21,133 | 5,376 | 139,276 |
| Avg. read length (bp) | 98 | 799 | 1040 |
| Putative CDSs | 8,558 | 4,640 | 142,996 |
| Total subsystem assignments | 5,723 | 5,127 | 174,711 |
| Total number of subsystems | 406 | 448 | 594 |

^*^ Treusch et al., 2004

^**^Tringe et al., 2005

Details of MG-RAST analysis of putative mRNA-tags and two soil metagenome datasets used in functional comparison to the mRNA-tags are given.
