# Supplementary material for: Simultaneous Assessment of Soil Microbial Community Structure and Function through Analysis of the Meta-Transcriptome
Source: PLoS One. 2008 Jun 25;3(6):e2527. doi: 10.1371/journal.pone.0002527 (PMC2424134; doi:10.1371/journal.pone.0002527)
Supplement: Table S9 — Functional subsystems with significantly higher abundance in putative mRNA-tags compared to a metagenomic sample. (0.03 MB DOC) [file pone.0002527.s019.doc]

**Supplementary Table ST9:** Functional subsystems with significantly higher abundance in putative mRNA-tags compared to the metagenomic sample collected earlier from the same site (Treusch et al, 2004), identified as described from Rodriguez-Brito et al., 2006.

| Subsystem |
| --- |
| Fe-S cluster assembly |
| GroEL GroES |
| Polyadenylation bacterial |
| Proteasome bacterial |
| Proteolysis in bacteria, ATP-dependent |
| Ribosome LSU bacterial |
| Ribosome SSU bacterial |
| Ribosome SSU chloroplast |
| RNA polymerase bacterial |
| Tetracycline resistance, ribosome protection type |
| Translation factors bacterial |
| Universal GTPases |
